# A sequential integrating virtual simulation and authentic video for preschool teacher candidates’ observational skills training: A design-based intervention study

**DOI:** 10.1371/journal.pone.0358951

**Published:** 2026-09-21

**Authors:** Beibei Zhang, Shizhu Gou, Deqiang Wang, Xingjian Zhu, Yuhui Han, Yaqi Sun, Ying Li, Qinglei Hu, Xueying Liu

**Affiliations:** 1 School of Teacher Education, Hebei Normal University, Shijiazhuang, China; 2 School of Home Economics, Hebei Normal University, Shijiazhuang, China; 3 Institute of Early Childhood Education, Faculty of Education, East China Normal University, Shanghai, China; 4 The Second Kindergarten Affiliated to Hebei Normal University, Shijiazhuang, China; Farhangian Teacher Education University: Farhangian University, IRAN, ISLAMIC REPUBLIC OF

## Abstract

Professional observation ability is a foundational skill for preschool teachers, yet its cultivation has long faced the challenge of bridging theory and practice. Virtual simulation and authentic video offer new technological pathways to address this gap, but research on their sequential integration remains limited. This study adopted a design-based research-informed intervention approach, conducting four sequential workshops over six weeks with one natural class of 46 preschool teacher candidates. A mixed-methods design was employed, integrating group observation assignment scores (32 valid samples), questionnaires (34 students), and semi-structured interviews (9 students). In the initial skill acquisition phase, virtual simulation and authentic video demonstrated functional complementarity: virtual simulation was more conducive to stimulating active thinking (purposefulness dimension, p = 0.006), while authentic video showed an advantage in honing detail-capturing abilities (acuity dimension, p = 0.048) and higher-order cognitive skills (analysis and support dimensions, p < 0.05). During sequential exposure to authentic videos, analytical ability showed a “leap-plateau” pattern, supportive ability displayed continuous growth, while basic observation ability remained stable. Learners’ experiences followed a trajectory from cognitive conflict to support seeking and then to metacognitive growth. This study provides preliminary support for the sequential integration model in enhancing preschool teacher candidates’ observational skills, offering empirical insights for technology-enhanced teacher education. The findings suggest that teacher training programs should consider the complementary roles of different technological media across skill development stages.

## 1. Introduction

Under the global consensus of the United Nations Sustainable Development Goal 4 (SDG 4), which aims to “ensure inclusive and equitable quality education and promote lifelong learning opportunities for all” [1], teacher quality is recognized as a core element for achieving quality education [2]. This is particularly critical in early childhood education, where teachers’ professional competence directly impacts children’s developmental trajectories during a crucial period of growth [3].

Among the professional competencies required of teachers, professional observation ability—the capacity to purposefully perceive, interpret, and respond to pedagogical events in complex classroom situations—is recognized as a foundational skill [4]. As Sherin and van Es argue, this “professional vision” is a key mediator in transforming theoretical knowledge into effective teaching practice, determining whether a teacher can capture crucial information amidst complex classroom dynamics, understand the meaning behind children’s behaviors, and make appropriate pedagogical responses accordingly [5].

While observation is important for all teachers, preschool teachers face unique challenges that make this skill particularly demanding. Unlike primary or secondary school teachers, preschool educators work with children who express themselves predominantly through non-verbal cues, spontaneous actions, and ambiguous behaviors rather than through structured verbal responses [6]. Preschool teachers must simultaneously monitor multiple children engaged in parallel activities, interpret behavioral signals without relying on academic task performance, and make split-second pedagogical decisions—all without the framing provided by formal lesson structures [7]. This complexity makes observational skills not merely an asset but a prerequisite for effective practice. However, preservice preschool teachers typically enter training with limited experience in systematic observation and often find it overwhelming to attend to multiple children’s behaviors simultaneously [8].

Effectively cultivating this core ability among preschool teacher candidates (TCs) remains a persistent challenge. Traditional teacher training models are often criticized for the theory-practice gap: students learn abstract theories in university classrooms but feel overwhelmed when entering real kindergarten settings [9]. Korthagen terms this the “theory-practice gap,” noting its roots lie in separating theoretical knowledge from clinical practical experience [10]. This disconnect is particularly detrimental in preschool contexts, where the gap between theoretical principles and the unpredictable, moment-to-moment reality of classroom interactions is exceptionally wide [11].

### 1.1. Technology-enhanced teacher education: Two mainstream Approaches

Information and Communication Technology (ICT) offers new possibilities for bridging this gap. Two approaches have gained prominence: authentic video training and virtual simulation training.

Authentic Video Training. Classroom video analysis is widely used due to its high ecological validity. By viewing real teaching footage, TCs can access authentic practice and connect theory to real-life contexts [12]. However, the complexity of authentic classrooms is a double-edged sword. For novice teachers, rich contextual information can create excessive cognitive load, making it difficult to focus on key pedagogical events [13]. Without scaffolding, novice teachers may be overwhelmed and struggle to extract meaningful learning [5].

Virtual Simulation Training. In contrast, virtual simulations offer a controllable, repeatable practice environment where complexity can be adjusted to learners’ levels [14]. Research shows these environments can reduce extraneous cognitive load, allowing learners to focus on core skills [15]. Badilla-Quintana and Sandoval-Henríquez found that virtual environments, through clear interfaces and manipulable objects, foster students’ sense of interactivity, presence, and flow [16]. However, the simplification that enables these benefits also means simulations may not fully replicate the complexity and dynamism of real classrooms, and their authenticity is inherently limited [17].

### 1.2. Research gap: From media comparison to sequential Integration

Reviewing existing literature, most studies focus on the immediate effects of a single medium (either AV or virtual simulation) in teacher training [12,14]. For example, Gaudin and Chaliès’ [12] systematic review synthesizes evidence on video-based teacher learning, while Dieker et al. [15] and Badilla-Quintana and Sandoval-Henríquez [16] examine the affordances of virtual simulation environments for preservice teachers. Recent systematic reviews further confirm that virtual reality simulations in teacher education primarily target affective outcomes such as self-efficacy, with interventions typically short in duration and lacking longitudinal tracking of skill transfer [18,19]. These studies provide a foundation for understanding the value of each technology but reveal two key gaps.

First, there is a lack of exploration of longitudinal integration pathways. Existing research largely consists of A vs B media comparisons—for instance, Seidel et al. [13] compared teachers‘own teaching videos versus others’ videos; Huang et al [20]. compared VR simulation versus video tasks in classroom management training; and Badilla-Quintana and Sandoval-Henríquez [16] examined virtual world interventions without incorporating authentic video. While these comparisons are informative, they focus on which single technology is superior rather than how different media might be sequenced across a developmental trajectory. However, as teacher professional development theories indicate, teacher growth is a continuous, staged process. Berliner’s model of teacher professional development stages suggests that the journey from novice to expert requires accumulation and progression through distinct phases [21]. Therefore, what is needed is not determining the superiority of “virtual” over “real”, but exploring how both can be integrated sequentially across the continuum of teacher development to achieve complementary advantages. To date, studies that explicitly design and evaluate sequential or blended technology integration models—where virtual simulation is used as an initial scaffold and authentic video is introduced progressively—remain notably absent in the literature.

Second, most existing studies focus on the immediate effects of training, while overlooking the long-term, retention and transferability of acquired skills. Systematic reviews have noted that VR-based teacher training interventions are typically short and infrequent, with overreliance on self-report measures and a lack of in-simulation assessment or longitudinal tracking of skill transfer [18,19]. Teacher learning is a cumulative process, and the extent to which initial training benefits translate into sustained competence in real classrooms remains under‑investigated [22,23]. It is therefore unclear whether and how these two tools-virtual simulation and AV, each with distinct strengths-can be sequentially integrated within a long-term, systematic training program to support the gradual development of teacher competence.

## 2. Theoretical framework: A scaffolding-transfer integration model

To address the research gap outlined above, this study integrates three theoretical perspectives to construct a Scaffolding-Transfer teacher observational skill development model, serving as the theoretical framework guiding research design and result interpretation.

First, Cognitive Load Theory (CLT). Drawing on Sweller’s CLT [23], this study positions virtual simulation videos (VS) as a cognitive scaffold in the initial phase of skill acquisition. This theory posits that learners’ working memory capacity is limited. In the initial phase of skill acquisition, excessive extraneous cognitive load—that is, the cognitive burden imposed by poor instructional design or the task environment itself-hinders effective schema construction [24]. Therefore, using virtual simulation environments to streamline and purify complex teaching situations can effectively reduce beginners’ extraneous cognitive load, allowing their valuable cognitive resources to focus on identifying and interpreting core observation targets. This establishes the foundation for subsequent deep learning.

Second, Situated Learning Theory (SLT). Drawing on Lave and Wenger’s SLT [25], this study positions AV as a vehicle for practice in the phase of skill deepening and transfer. This theory emphasizes that learning is inherently social and situated; the meaning of knowledge is constructed through interaction with authentic contexts. Once learners have mastered basic observational skills, they need to be embedded in authentic, diverse practical contexts to develop adaptive expertise capable of flexibly handling uncertainty-through participation in a practical community. AV sequences serve as an ideal medium for presenting such complex practical contexts. This is the key to achieving successful transfer of knowledge and skills from the training setting to the real classroom.

Based on the theoretical integration outlined above, this study proposes the Scaffolding-Transfer Integration Framework for teacher observational skill development. This framework divides the training process into two sequential yet organically connected phases: Phase 1 (Focus Foundation), where learners need robust technological scaffolding; the Virtual Simulation environment plays this role by simplifying contexts and focusing on core elements, helping learners establish basic observation schemas under low cognitive load. Phase 2 (Deepening Transfer), where, after learners possess preliminary observational abilities, they require authenticity challenges; carefully selected and organized AV sequences provide diverse contexts ranging from simple to complex and typical to atypical, driving learners to continuously apply, test, and refine their observational skills within a real problem space.

The framework was operationalized as follows. In the VS workshop, design features were aligned with CLT principles by pre-structuring the video scenario with clear narrative cues, minimizing extraneous details such as side conversations or overlapping activities, and providing observation prompts that directed attention to focal children. In the AV workshops, design features were aligned with SLT principles by presenting unscripted, naturally occurring classroom interactions, progressively increasing contextual complexity across workshops, and requiring students to identify relevant events without pre-structuring.

This model aims to achieve a smooth transition from scaffolding to fading through the sequential integration of technology and context, ultimately fostering the long-term development of teachers’ professional competence.

Based on the theoretical framework and identified research gap, this study adopts a design-based intervention approach to explore the sequential integration pathway of virtual simulation and authentic video in developing preschool TCs’ observational skills. Specifically, this study addresses the following three research questions:

RQ1:In the initial skill acquisition phase, what are the differential effects of Virtual Simulation and AV on TCs’ observational skill performance and observation quality?

RQ2:How do TCs’ observational skills evolve as they sequentially engage with AV sequences progressing from typical to complex scenarios?

RQ3:How do learners perceive the value of this “Virtual Simulation-Authentic Video” sequential integration model for their professional growth?

## 3. Materials and methods

### 3.1. Institutional review board statement

This study was approved by the University Committee on Human Research Protection at East China Normal University (Approval No. HR178‑2019). The approval remained valid at the time of data collection and specifically permitted the use of classroom video recordings for research purposes, with children’s faces anonymized. The research participants were TCs majoring in early childhood education, all of whom were adults with full legal capacity. All participants decided whether to participate in the survey based on their own voluntary will, after being fully informed of the research purpose and procedures. Those who agreed to participate proceeded to complete the questionnaire, while those who did not agree could close the questionnaire link at any time. All interview content was used with the consent of the participants and was anonymized during presentation to protect participant privacy.

### 3.2. Participants

This study selected one natural class of students majoring in early childhood education from a university as in northern China the research participants. The class consisted of 46 students, including 38 females and 8 males. This was a non-random, intact-class sample drawn from a single institution, which is appropriate for an exploratory intervention study but limits the generalizability of the findings.

During the course learning process, the students were divided into 9 fixed groups according to their daily grouping habits. The research was conducted within the framework of their specialized course, Child Behavior Observation, and involved four sequential workshop activities integrated with the course content over a six-week teaching cycle (two sessions per week, totaling 90 minutes per week).

**Fig 1:**
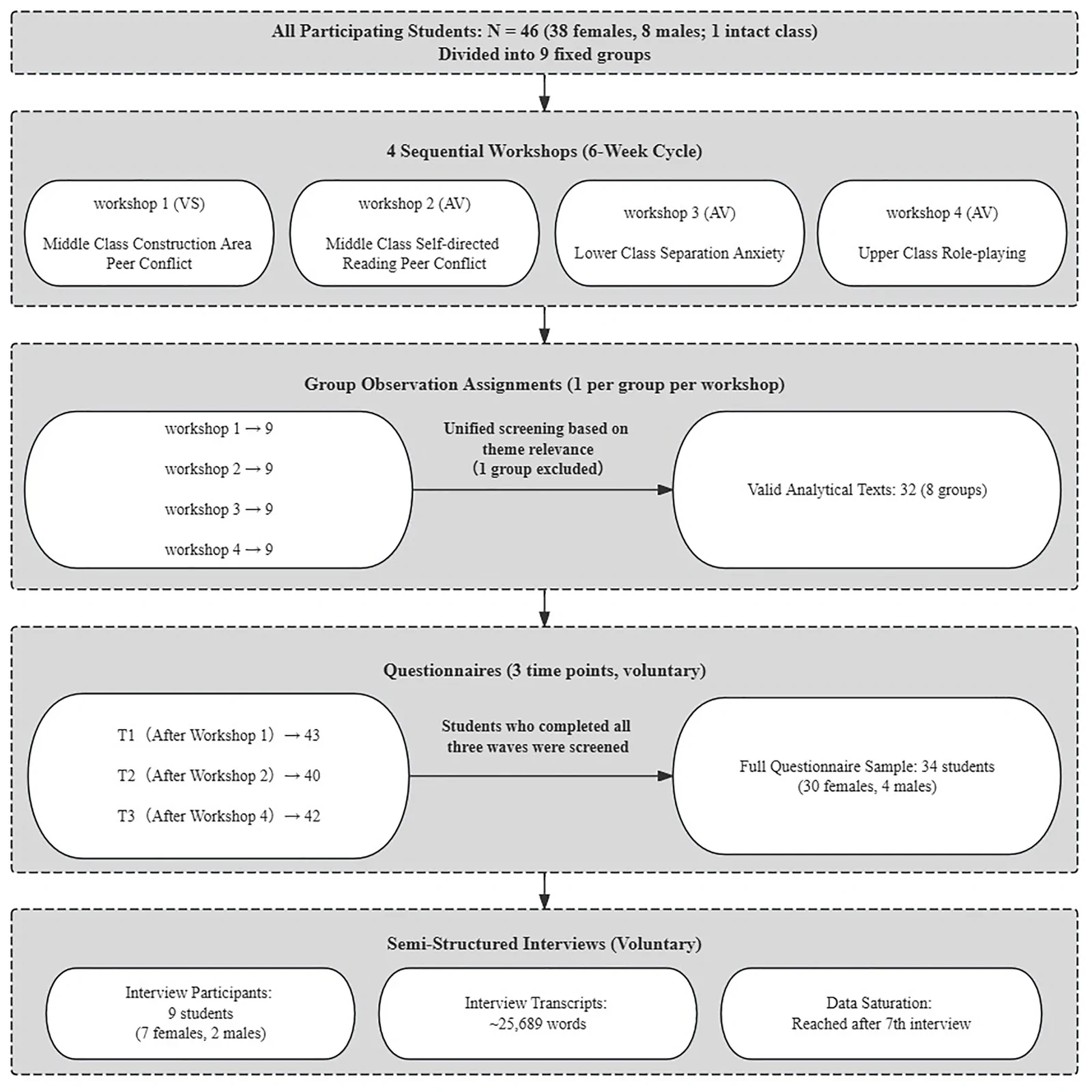
Participant flow diagram. The diagram illustrates the number of students and groups contributing to each type of data at each stage of the study. VS = Virtual Simulation; AV = Authentic Video; T1, T2, and T3 represent the three questionnaire time points (after Workshop 1, Workshop 2, and Workshop 4, respectively). Group-level data: 9 groups submitted observation assignments after each workshop (36 submissions in total); one group’ s data were excluded due to misalignment with the workshop theme, yielding 32 valid analytical texts (8 groups × 4 workshops). Individual-level data: 34 students completed all three questionnaire waves (30 females, 4 males); 9 students (7 females, 2 males) participated in semi-structured interviews after all workshops. Data saturation was reached after the seventh interview.

At the time of the study, none of the participants had prior experience with virtual simulation or systematic video analysis training. However, during the semester, all students had completed a one-day kindergarten observation practicum as part of their program’s required fieldwork, providing them with basic familiarity with classroom contexts. A participant flow diagram illustrating the number of students contributing to each type of data at each stage is provided in Fig 1.

### 3.3. Research design and procedure

This study adopted a design-based intervention approach, aiming to explore the sequential integration pathway of VS and AV in observational skills training for TCs in early childhood education. Although the study adopted a DBR orientation, the research team’s iterative refinements were primarily implemented between workshop cycles based on ongoing observations of student performance and informal feedback. However, due to the fixed six-week course structure, substantial redesigns were not feasible within the implementation period. Therefore, we describe this study as DBR-informed rather than a full DBR study.

Before the workshops commenced, the research team (comprising university faculty, graduate students, and a kindergarten principal) conducted thematic discussions focused on typical behavioral manifestations across different age groups in kindergarten, from nursery classes to senior classes, and subsequently determined the themes for the four workshops. The workshop themes progressed sequentially according to the developmental order of children’s ages and the increasing complexity of behavioral contexts. A summary of the four workshops is provided in Table 1.

**Table 1:** Summary of the four sequential workshops.

Workshop	Workshop 1	Workshop 2	Workshop 3	Workshop 4
**Video Type**	Virtual Simulation (VS)	Authentic Video (AV)	Authentic Video (AV)	Authentic Video (AV)
**Duration**	3-5 min			
**Age Group**	Middle class	Middle class	Lower class	Upper class
**Theme/Context**	Peer conflict in the block construction area	Peer conflict during self-directed reading	Separation anxiety	Role-playing
**Learning Objectives**	Establish basic observation schemas under low cognitive load, focusing on core behavioral identification	Initiate exposure to authentic contexts; apply observation skills to unscripted scenarios	Achieve a breakthrough in analytical competence through thematic transition	Integrate observation, analysis, and support competencies in complex social contexts
**Observation Prompts**	Focus on conflict triggers, children’s behavioral expressions, and teacher intervention strategies	Independently identify observation targets, behavioral events, and their developmental trajectories	Focus on children’s emotional expressions, attachment behaviors, and teacher soothing strategies	Focus on peer interactions, role assignment, social engagement, and teacher support strategies
**Group Activity Flow**	View video → Group discussion (20 min) → Write analytical text			
**Instructor Feedback**	Three-dimensional feedback (theoretical/historical/environmental)			
**Assignment Requirements**	Complete text (observation + analysis + support)	Complete text (observation + analysis + support)	Analysis dimension only	Complete text (observation + analysis + support)
**Additional Support**	None	Frontline kindergarten teacher online sharing session (approx. 20 min)	None	None

**Note:** All videos were 3–5 minutes in duration. VS materials were independently developed by the research team using the Doubao (Doubao-Seed-3D-1.0) large language model (ByteDance), which supports end-to-end generation of simulation-grade 3D models from textual prompts. AV materials were provided by a collaborating kindergarten (recorded in daily classroom settings), with children’s faces anonymized using mosaic processing. The workshop themes were designed to progress according to children’s age levels and increasing contextual complexity.

**Fig 2:**
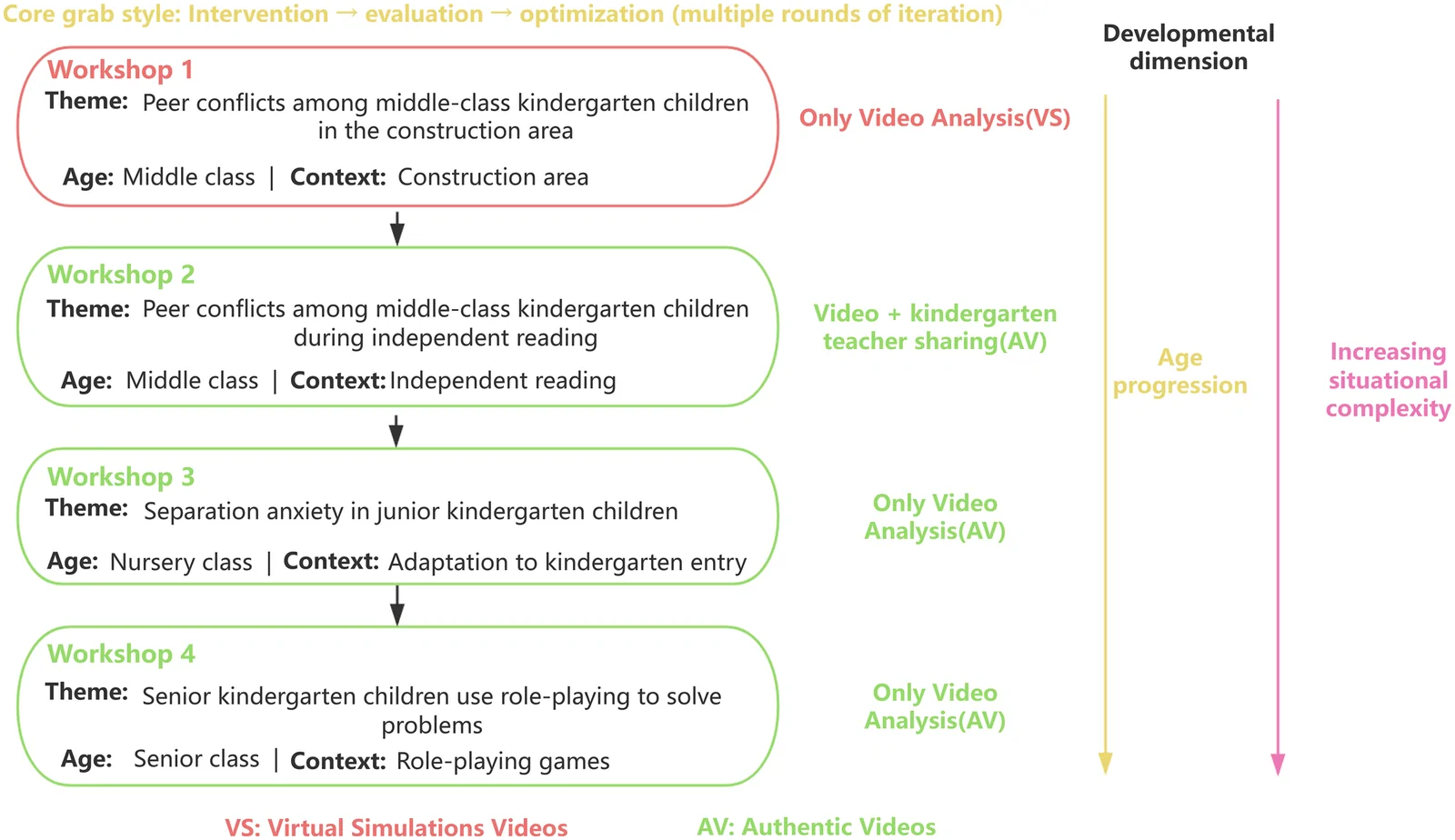
Overview of the four sequential workshops in the design-based study.

The sequential design of the four workshops is illustrated in Fig 2. It should be noted that Workshop 2 included an additional online sharing session delivered by a frontline kindergarten teacher. This extra support may have influenced student performance in the AV condition and is acknowledged as a potential confounding factor in the Discussion and Limitations sections (see Sections 5.1 and 6.2).

### 3.4. Research materials and instruments

#### 3.4.1. Video materials.

In accordance with the themes of each workshop, the research team independently developed VS materials (used in Workshop 1) using the Doubao (Doubao-Seed-3D-1.0) large language model developed by ByteDance, which supports end-to-end generation of simulation-grade 3D models from textual prompts. The AV were provided by the kindergarten principal who was a member of the research team, using footage recorded in the kindergarten’s daily classroom settings (used in Workshops 2, 3, and 4). Each video lasted approximately 3–5 minutes and was selected based on the clarity of behavioral episodes relevant to each workshop’s theme. To safeFguard the rights and privacy of minors, mosaic processing was applied to the faces of children in all AV. We acknowledge that the video quality, particularly in the VS condition, was not flawless; students’ perceptions of video quality are reported as part of the qualitative findings in Section 4.3.1.

#### 3.4.2. Group observation assignments.

Following each workshop, the 9 groups were required to submit a group observation assignment, with the content focusing on analysis related to the theme of the respective workshop. Among these submissions, the materials submitted for the third workshop primarily consisted of “analysis dimensions”, while the other submissions were comprehensive (including observation dimensions, analysis dimensions, and support dimensions). To ensure the quality of the analytical materials, the research team screened the submissions based on the degree of alignment between the submitted content and the workshop themes, resulting in the exclusion of one set of data. Ultimately, 32 valid analytical texts were obtained.

To quantify the analytical quality of students’ observation texts, the research team developed a detailed scoring rubric based on the three core dimensions involved in child behavior observation-“observation, analysis, and support”:

Observation Dimension (40 points): This dimension assessed whether students could objectively and accurately describe children’s behaviors, while attending to contextual factors such as the physical environment, peer interactions, and teacher involvement.

Analysis Dimension (30 points): This dimension assessed whether students could interpret children’s behaviors by applying relevant theories, such as those related to child development and psychology.

Support Dimension (30 points): This dimension assessed whether students could propose actionable educational support suggestions by integrating contextual information such as children’s prior experiences and developmental needs.

The complete scoring rubric, including descriptors for each performance level, is provided in S1 Table.

The scoring was conducted independently by two researchers without interference between them. Both raters participated in a two-hour training session using five practice texts to calibrate scoring standards. All scoring was conducted blind to workshop condition. Disagreements were resolved through discussion; if consensus could not be reached, the two raters consulted a third researcher. Consistency checks were performed on 20% of the scored texts midway through the process. Inter-rater reliability analysis revealed that the average-measures intraclass correlation coefficient (ICC) was 0.753 (95% confidence interval: 0.494–0.880,p < 0.001), indicating a good level of consistency between the two raters [26]. The wide confidence interval is acknowledged as a limitation (see Section 6.2). Accordingly, the average score of the two raters was used as the final score for each group observation assignment in subsequent analyses.

#### 3.4.3. Questionnaire survey.

Following discussions within the research team and drawing upon relevant literature [27–29], this study developed a self-administered questionnaire from the students’ perspective, titled the Self-Assessment Questionnaire on Observational Skills for Teacher Candidates. The items were developed based on a literature review of teacher observation competencies, and the content validity of the questionnaire was ensured through discussions within the research team.

The questionnaire comprised three dimensions-observation awareness, observation knowledge, and observation quality-with a total of 22 items. It adopted a 5-point Likert scale (1–5 representing “strongly disagree” to “strongly agree”, respectively). Students were invited to complete the questionnaire online after the first workshop, after the second workshop, and after all four workshops were concluded. The questionnaire was administered on a completely voluntary basis. A total of 43 valid questionnaires were collected after the first workshop, 40 after the second workshop, and 42 after all workshops were concluded. To ensure the validity of the longitudinal comparison, the study further integrated the three waves of data and screened out 34 students who had fully participated in all three questionnaire administrations as the core research sample (including 30 females and 4 males).

Due to the small sample size and the non-normal distribution of the data, non-parametric statistical methods (Wilcoxon signed-rank tests) were employed for the questionnaire data analysis to ensure the robustness of the statistical inferences. Reliability analysis was also conducted on the questionnaire. The Cronbach’s α coefficients for each dimension and the overall questionnaire ranged from 0.608 to 0.899. We acknowledge that the lower bound of this range falls in the marginally acceptable range, reflecting both the small sample and the exploratory nature of the instrument. Therefore, results from this questionnaire should be interpreted as preliminary [30].

#### 3.4.4. Interview method.

This study adopted a semi-structured interview protocol developed by the researchers, aiming to gain in-depth insights into students’ experiential feelings, perceived differences, and confusion during observations in virtual and authentic kindergarten contexts. The interview protocol mainly consisted of four parts:

1. Initial impressions of observing VS and AV;
2. Perceived differences between observing VS and AV;
3. Confusion encountered during observations in virtual and authentic contexts;
4. Suggestions regarding the arrangement of observations in virtual or authentic contexts.

During the interviews, the researchers posed flexible follow-up questions based on the students’ responses to further explore their authentic experiences. All interviews were conducted individually by the researchers through online voice calls, with an average duration of 20 minutes per interview.

Interviewees were recruited through voluntary sign-up after all workshops. We aimed for maximum variation in terms of gender and group membership. Data saturation was reached after the seventh interview, with two additional interviews conducted to confirm that no new themes emerged. Initially, 10 students were recruited, among whom one withdrew, resulting in a total of 9 interviewees (7 females and 2 males).

The interview process was audio-recorded in its entirety with the consent of the participants. Subsequently, all audio recordings were transcribed verbatim into text. After verification and organization, a raw text corpus of approximately 25,689 words was ultimately formed. We acknowledge that no formal memo-writing procedures were implemented during this study. However, we addressed potential researcher subjectivity through several complementary mechanisms. First, the two coders independently analyzed the interview data before comparing their interpretations, and regular team discussions were held to examine whether coding decisions were grounded in participant responses rather than researchers’ prior assumptions. Second, when disagreements arose, a third researcher was consulted to provide an external perspective. Third, during the analysis process, the research team documented emerging interpretations and engaged in debates over alternative readings of participant responses. Although we did not produce formal written memos, these reflexive dialogues within the research team functioned as a continuous quality-check mechanism to maintain analytic transparency. This study employed a reflexive thematic analysis approach to code the interview data. Two researchers conducted independent coding, after which the results were compared, and discrepancies were discussed to reach consensus. Where consensus could not be achieved, a third senior qualitative research scholar was invited to provide objective evaluation until a unified coding framework was established. Ultimately, a comprehensive coding system was developed, comprising three core themes (the challenge of finding meaning in chaos, the desire for structured support, and the awareness of one’s own growth), 9 cluster codes, 26 open codes, and 167 valid interview excerpts. This coding system fully captured the experiences and perceptions of TCs during observations in virtual and authentic contexts.

## 4. Results

### 4.1. Differences in initial effects of VS and AV on observational skills performance and observation quality

#### 4.1.1. Quantitative comparison of observational skills performance: based on group observation assignment scores.

To examine the differential effects of VS and AV on TCs observational skills performance in the initial stage of skill acquisition, this study conducted a paired-sample t-test on the scores of group observation assignments submitted after the first workshop VS and the second workshop AV. Due to the small sample size and the non-normal distribution of the data, Wilcoxon signed-rank tests were also conducted as a robustness check. For significant comparisons, effect sizes (Cohen’s d) and 95% confidence intervals for the mean differences are reported. The comparison indicators included the total score of the observation assignments, as well as the scores for the three core dimensions of “observation, analysis, and support”. The results are presented in Table 2.

**Table 2:** Comparison of observational skills: VS vs. AV.

	M	SD	Paired-Samples t-Test(t/p)	Cohen’s d	95% CI		Wilcoxon(z/p)
					Lower Bound	Upper Bound	
**VS Total Score**	81.75	2.75	−3.439 *(0.011)	−1.216	−2.126	−0.261	−2.375*(0.018)
**AV Total Score**	85.00	1.60					
**VS-Observation Dimension**	34.50	0.89	−1.861(0.105)	−0.658	−1.411	0.131	−1.620(0.105)
**AV-Observation Dimension**	35.38	0.74					
**VS-Analysis Dimension**	23.69	1.36	−3.480*(0.010)	−1.231	−2.145	−0.270	−2.328*(0.020)
**AV-Analysis Dimension**	24.75	0.89					
**VS-Support Dimension**	23.63	1.03	−2.546*(0.038)	−0.900	−1.712	−0.046	−1.983*(0.047)
**AV-Support Dimension**	24.88	0.99					

Note: *p < 0.05

In terms of overall performance, the total score of group observation assignments under the AV condition (M = 85.00, SD = 1.60) was significantly higher than that under the VS condition (M = 81.75, SD = 2.75), t = −3.439, p = 0.011 < 0.05, Cohen’s d = [−1.216], 95% CI for the mean difference [−2.126, −0.261]. The Wilcoxon signed-rank test yielded z = −2.375, p = 0.018 < 0.05, indicating that the results for this dimension are statistically robust. This result indicates that although the VS was designed as a scaffold to reduce initial cognitive load, the group exposed to the AV achieved higher scores in overall observation task performance. Further analysis of the scores across dimensions reveals that the differences were primarily reflected in the following aspects:

First, in terms of the observation dimension, the score under the VS condition was 34.50 ± 0.89, while that under the AV condition was 35.38 ± 0.74. Although there was a mean difference between the two, it did not reach statistical significance (t = −1.861, p = 0.105 > 0.05). This indicates that at the fundamental level of observation-namely, accurately describing children’s behaviors and attending to environmental and interactional details-there was no significant difference between the effects of VS and AV on student performance. Although the VS presented a simplified context, it did not weaken students’ performance in the basic observation dimension; meanwhile, despite the complexity of the context in the AV, students were still able to complete fundamental observation tasks with the support of the instructor.

Second, in terms of the analysis dimension, the score under the AV condition (24.75 ± 0.89) was significantly higher than that under the VS condition (23.69 ± 1.36), t = −3.480, p = 0.010 < 0.05, Cohen’s d = [−1.231], 95% CI for the mean difference [−2.145, −0.270]. The non-parametric Wilcoxon signed-rank test yielded consistent results (z = −2.328, p = 0.020 < 0.05), indicating that the findings for this dimension are statistically robust. This finding warrants attention. The analysis dimension assessed whether students could interpret children’s behaviors by applying relevant theories, such as those related to child development and psychology. Due to the complexity and richness of its context, the AV may have provided students with more behavioral details and interactional cues “worthy of analysis”, thereby stimulating deeper theoretical application and meaning-making. In contrast, while the simplified context of the VS helped reduce cognitive load, it may have, to some extent, narrowed the range of behavioral dimensions available for analysis, thereby limiting students’ opportunities for deeper engagement with theoretical content.

Third, in terms of the support dimension, the score under the AV condition (24.88 ± 0.99) was also significantly higher than that under the VS condition (23.63 ± 1.03), t = −2.546, p = 0.038 < 0.05, Cohen’s d = [−0.900], 95% CI for the mean difference [−1.712, −0.046]. The non-parametric Wilcoxon signed-rank test yielded consistent results (z = −1.983, p = 0.047 < 0.05), indicating that the findings for this dimension are statistically robust. The support dimension assessed whether students could propose actionable educational suggestions by integrating children’s prior experiences and developmental needs. This result may stem from the fact that the children’s behaviors presented in the AV were embedded in richer contextual information-students were able to observe more information from the video regarding individual children’s characteristics, classroom environment, and other relevant details, which provided a basis for proposing targeted educational support. While the VS was well-designed, its characteristic of lacking contextual information may have posed a certain limitation in terms of performance on the support dimension.

In summary, in the initial stage of skill acquisition, AV demonstrated significant advantages over VS in overall observation task performance, as well as in the two higher-order cognitive dimensions of analysis and support. This finding suggests that although VS has theoretical validity as a “supporting tool” in reducing cognitive load, the richness of authentic contexts may have stimulated students’ deeper cognitive processing at an earlier stage. However, this finding still needs to be interpreted in conjunction with subsequent qualitative data to gain a more comprehensive understanding of the mechanisms through which the two media function in the initial stage of skill acquisition.

#### 4.1.2. Quantitative Comparison of Observation Quality: Based on Self-Report Questionnaire Data.

To examine the differential effects of VS and AV on TCs observation quality in the initial stage of skill acquisition, this study conducted a paired-sample t-test on the scores of items related to the “observation quality” dimension in the questionnaires completed by the 34 core sample students after the first workshop VS and after the second workshop AV. Observation quality comprised seven sub-dimensions: purposefulness, organization, comprehensibility, accuracy, acuity, persistence, and originality. The results are presented in Table 3.

**Table 3:** Observational quality: VS vs. AV.

	M	SD	Paired-Samples t-Test	Cohen’s d	95% CI		Wilcoxon(z/p)
					Lower Bound	Upper Bound	
**Purposefulness (X)**	4.26	0.51	2.956*(0.006)	0.507	0.146	0.861	−2.668*(0.008)
**Purposefulness (Z)**	3.94	0.65					
**Organization (X)**	3.82	0.76	−1.139(0.263)	−0.195	−0.533	0.146	−1.062(0.288)
**Organization (Z)**	4.00	0.65					
**Comprehensibility (X)**	4.03	0.49	−0.725(0.473)	−0.124	−0.461	0.214	−0.693(0.489)
**Comprehensibility (Z)**	4.09	0.53					
**Accuracy (X)**	3.76	0.96	0.177(0.861)	0.030	−0.306	0.366	−0.131(0.896)
**Accuracy (Z)**	3.74	0.93					
**Acuity (X)**	3.59	0.82	−2.055*(0.048)	−0.352	−0.697	−0.003	−1.937(0.053)
**Acuity (Z)**	3.85	0.56					
**Persistence (X)**	3.26	1.05	0.000(1.000)	0.000	−0.336	0.336	−0.189(0.850)
**Persistence (Z)**	3.26	1.05					
**Originality (X)**	3.62	0.78	−0.796(0.432)	−0.137	−0.473	0.202	−0.743(0.457)
**Originality (Z)**	3.76	0.82					

Note: *p < 0.05

Due to the small sample size and the non-normal distribution of the data, non-parametric Wilcoxon signed-rank tests were also conducted as a robustness check. Effect sizes (Cohen’s d) and 95% confidence intervals for the mean differences are reported for significant comparisons.

The data showed that in the purposefulness dimension, the self-reported score of students after the VS (M = 4.26, SD = 0.51) was significantly higher than that after the AV (M = 3.94, SD = 0.65), t = 2.956, p = 0.006 < 0.01, Cohen’s d = [0.507], 95% CI for the mean difference [0.146, 0.861]. The non-parametric Wilcoxon signed-rank test yielded consistent results (z = −2.668, p = 0.008 < 0.01), indicating that the findings for this dimension are statistically robust. The purposefulness dimension corresponded to Item 15: “During observation, I reflect on the phenomena and problems that arise in the context.” This result indicates that under the VS condition, students were more inclined to actively reflect on the reasons and problems underlying the phenomena during the observation process. A possible explanation is that the relatively simplified context and clear cues of the VS reduced students’ cognitive load in information filtering, allowing them to allocate more attentional resources to deeper reflection on the phenomena. In contrast, due to the high density of information and contextual complexity of the AV, students may have needed to devote more effort to “understanding what happened” rather than “reflecting on why it happened”.

In the acuity dimension, the self-reported score of students after the AV (M = 3.85, SD = 0.56) was significantly higher than that after the VS (M = 3.59, SD = 0.82), t = −2.055, p = 0.048 < 0.05, Cohen’s d = [−0.352], 95% CI for the mean difference [−0.697, −0.003]. The non-parametric Wilcoxon signed-rank test, however, did not reach statistical significance (z = −1.937, p = 0.053 > 0.05), suggesting that the robustness of this finding should be interpreted with caution. The acuity dimension corresponded to Item 20: “During observation, I am able to quickly notice subtle changes in children’s emotions or behaviors.” This result presents an interesting contrast to the finding in the purposefulness dimension: although the AV may have weakened students’ inclination toward active reflection, it significantly enhanced their sensitivity to details. This may be because AV presented richer, more natural behaviors, compelling students to maintain heightened alertness to capture key information. In contrast, the prescriptiveness of VS may have reduced students’ perceived need for such sensitivity.

In contrast, there were no significant differences in self-reported scores between the VS and AV conditions in the five dimensions of organization (p = 0.263), comprehensibility (p = 0.473), accuracy (p = 0.861), persistence (p = 1.000), and originality (p = 0.432).

It can be seen that in the initial stage of skill acquisition, VS and AV exerted differential effects on students’ observation quality: VS was more conducive to stimulating students’ active reflection during observation (purposefulness), whereas AV showed a tendency to enhance detail sensitivity (acuity), although the robustness of this finding was insufficient. This finding echoes the result in Section 4.1.1 that “AV scored significantly higher in the analysis dimension”-AV not only promoted students’ analytical competence in terms of objective performance, but also enhanced their subjective sensitivity to details. Meanwhile, although VS did not demonstrate a significant advantage in objective performance, it provided students with an observational space more conducive to “focused reflection” at the subjective level. The two media thus demonstrated complementary potential in cultivating observation quality, providing a basis for subsequent sequential integration design.

#### 4.1.3. Qualitative evidence of observation quality: Based on interview data.

To corroborate and supplement the differences in observation quality revealed by the questionnaire data, this section extracts student statements related to each sub-dimension of observation quality from the interview data, presenting a comparison of students’ experiences with the two media-VS and AV. The following qualitative findings represent participants’ subjective perceptions and experiences. They are presented not as direct evidence of actual skill improvement, but as complementary evidence that helps interpret and contextualize the quantitative patterns observed.

4.1.3.1. Purposefulness and Comprehensibility: VS Prompts Thinking, AV Prompts Reflection: In terms of the purposefulness dimension (actively reflecting on phenomena and problems during observation), the interview data revealed that VS as more conducive to focused thinking due to its clear cues and fewer distractions. Student S1 mentioned: “With that AI video, the sequence of events was very clear, and I could intuitively see what the teacher should do or how things should go.” This clear presentation enabled students to focus their attention on thinking about the teaching events.

In terms of the comprehensibility dimension (connecting theory with practice and recognizing the limitations of theory), AV demonstrated unique advantages. Student S6’s statement was particularly representative: “Through observation and kindergarten fieldwork, I realized that there is a gap between the knowledge I learned and actual practice [...] It was not until after the fieldwork that I discovered the discrepancy between theory and real teaching contexts, and I gained a deeper understanding of the importance of integrating theory with practice.” This finding aligns with the trend observed in the questionnaire data, in which the comprehensibility score after AV was slightly higher (though not statistically significant). Student S1 further pointed out the difference between the two media in facilitating understanding: “When watching the AI VS, I tended to focus on the teacher’s role—how the teacher responded to children’s behaviors. When watching the AV, I paid more attention to children’s performance and feedback. Through this comparison, I gained a clearer understanding of the teacher’s role as a guide and responder in children’s activities.”

4.1.3.2. Acuity and Accuracy: AV Hones the Ability to Capture Details: In terms of the acuity dimension (quickly noticing subtle changes in children), the interview data consistently supported the advantage of AV. Student S8’s account reflected her perceived growth: “Watching AV allows me to observe better and more carefully. At first, I didn’t pay attention to small details; I just wanted to outline the general framework. Later, influenced by my group members, I started to notice more details, and my observational skills improved.” Student S8 also noted: “AV require me to summarize on my own. Unlike VS, which are simple and clear, I might need to watch an AV several times before I can figure out the whole story.” This process of repeated viewing and careful differentiation is precisely the mechanism through which acuity is honed.

In terms of the accuracy dimension (the gap between observation and ideal imagination), Student S6’s statement provided direct evidence: “I had always been listening to teachers talk about theories and watching videos, without having face-to-face interactions with children. It was not until after the fieldwork that I realized there is a discrepancy between theory and real teaching contexts.” This awareness of the “gap between ideal and reality” was reflected in both the VS and AV conditions, consistent with the finding of no significant difference in accuracy scores in the questionnaire data.

4.1.3.3. Persistence: AV Strengthens Professional Identity: In terms of the persistence dimension (enhancing professional identity and aspiration), the interview data revealed that observation through AV had a more profound impact on students’ professional cognition. Student S2 mentioned: “Observing real contexts is very important. It helps prepare us for future work and internships. When working in a kindergarten in the future, we need to teach while observing what is happening. Observing authentic contexts allows us to adapt to this situation in advance.” Student S5 further noted: “Observing authentic contexts helps improve our ability to respond to unexpected situations [...] This also reminds me that as a future early childhood teacher, I need to enhance my ability to handle unexpected situations.” This anticipatory thinking about future professional roles reflects the positive impact of observation activities on professional identity.

Overall, the interview data and questionnaire data formed a strong corroborative relationship. This mutual validation enhanced the reliability of the research findings: VS provided students with an observational space conducive to active thinking, while AV demonstrated unique value in honing the ability to capture details, deepening understanding of practice, and strengthening professional identity. The complementarity of the two media in cultivating observation quality provides a basis for subsequent sequential integration design.

### 4.2. Development of Observation Skills During Sequential Exposure to Diverse AV

#### 4.2.1. Longitudinal Evolution of Observation Skill Scores.

8 groups showed changes in their observational skills scores across the three dimensions of “observation” “analysis” and “support” after the second (conflicts during independent reading in the middle class), third (separation anxiety in the lower class), and fourth (role-playing in the upper class) workshops. It should be noted that, for the “Observation” and “Support” dimensions, due to differences in the workshop themes, scores for these two dimensions were compared only between the second and fourth sessions, where the themes were similar. For the “Analysis” dimension, as a core cognitive component of observational skills, complete data were available across all three workshops, enabling three longitudinal comparisons. The specific results are presented in Table 4. Given the small sample size, non‑parametric Wilcoxon signed‑rank tests were conducted as robustness checks. The results were consistent with the parametric tests. Effect sizes (Cohen’s d) and 95% confidence intervals are reported for significant comparisons.

**Table 4:** Longitudinal trends in observational skills.

	M	SD	Paired-Samples t-Test	Cohen’s d	95% CI		Wilcoxon(z/p)
					Lower Bound	Upper Bound	
**2-Observation**	35.38	0.74	0.513(0.623)	0.182	−0.524	0.874	−0.425(0.671)
**4-Observation**	35.19	0.80					
**2-Analysis**	24.75	0.89	T22 - T32, -6.093*(<0.001) T22 - T42, −2.550*(0.038) T32 - T42,0.077(0.941)	T22 - T32, -2.154	−3.439	−0.833	−2.524*(0.012)
**3-Analysis**	25.84	0.51		T22 - T42,-0.902	−1.714	−0.047	−1.904(0.057)
**4-Analysis**	25.81	0.84		T32 - T42,0.027	−0.667	0.719	−0.771(0.441)
**2-Support**	24.88	0.99	−3.230*(0.014)	−1.142	−2.027	−0.212	−2.226*(0.026)
**4-Support**	25.81	0.53					

Note: *p < 0.05

In terms of the observation dimension, the score after the second workshop was 35.38 ± 0.74, while the score after the fourth workshop was 35.19 ± 0.80, with no significant difference between the two (t = 0.513, p = 0.623 > 0.05). This finding indicates that after three sessions of sequential training with AV observations, the students’ fundamental observation skills did not show a significant improvement. A possible explanation is that the competencies assessed in the observation dimension had largely been established during the early stage of skill acquisition (with a score of 34.50 for this dimension after the first VS, increasing to 35.38 after the second AV), and subsequent training served more to consolidate and refine these skills rather than to achieve a quantitative leap. Additionally, the observation dimension score showed a slight (though non-significant) decline during the fourth session, which may be attributed to the increased contextual complexity of the fourth workshop’s theme, “Role-Playing in Large Classes”, where behavioral cues were more dispersed, thereby increasing the difficulty of observation.

Based on the analysis dimension, the scores across the three workshops exhibited a pattern of significant increase followed by stabilization. Specifically, the analysis dimension score after the second workshop was 24.75 ± 0.89, which significantly increased to 25.84 ± 0.51 after the third workshop (t = −6.093, p < 0.001, Cohen’s d = [−2.154], 95% CI for the mean difference [−3.439, −0.833]. The non-parametric Wilcoxon signed-rank test yielded consistent results (z = −2.524, p = 0.012 < 0.05), indicating that the findings for this dimension are statistically robust. A significant improvement was also observed between the second and fourth workshops (t = −2.550, p = 0.038 < 0.05, Cohen’s d = [−0.902], 95% CI for the mean difference [−1.714, −0.047]). The Wilcoxon signed-rank test yielded inconsistent results (z = −1.904, p = 0.057 > 0.05), indicating that the robustness of this dimension’s difference is insufficient. However, no significant difference was found between the third and fourth workshops (t = 0.077, p = 0.941 > 0.05).

This finding warrants further in-depth interpretation. The analytical dimension examined the students’ ability to explain children’s behavior using theories related to child development, psychology, and other relevant frameworks. The data show that, after their first exposure to AV, the students’ analytical skills were still at a relatively low level. However, after completing the second workshop, students’ analytical competence showed a qualitative leap. Although the improvement from the second to the fourth workshop was significant in the paired-samples t-test (p = 0.038), the Wilcoxon test did not reach statistical significance (p = 0.057), indicating that this difference lacked sufficient statistical robustness and should be interpreted with caution. Considering the consistently significant leap between the second and third workshops, and the absence of a significant difference between the third and fourth workshops (p = 0.941), it is reasonable to infer that the core improvement in analytical competence primarily occurred between the second and third workshops—that is, after students were first exposed to authentic video materials and completed observation training on the theme of separation anxiety. The less robust significance between the second and fourth workshops more likely reflects minor fluctuations in scores following the third workshop rather than sustained growth in analytical ability. Overall, the development of analytical competence followed a “rapid leap followed by stable maintenance” pattern, consistent with the typical trajectory of skill acquisition in which rapid early progress is followed by a consolidation phase. This pattern may also have been facilitated by the teacher sharing session after the first AV exposure and the accumulated practice across workshops.

In terms of the support dimension, the score after the second workshop was 24.88 ± 0.99, which significantly increased to 25.81 ± 0.53 after the fourth workshop (t = −3.230, p = 0.014 < 0.05, Cohen’s d = [−1.142], 95% CI for the mean difference [−2.027, −0.212]. The non-parametric Wilcoxon signed-rank test yielded consistent results (z = −2.226, p = 0.026 < 0.05), indicating that the findings for this dimension are statistically robust. The support dimension examines whether students can propose practical educational recommendations that align with young children’s experiences and developmental needs. This significant improvement suggests that as students accumulate experience in observing AV, they gradually shift from simply “observing” and “analyzing” toward a mindset of educational practice focused on “how to support” student learning. This shift is critical, as it marks the moment when students begin to translate the results of their observations and analyses into the basis for educational action. It is noteworthy that the trajectory of improvement in the support dimension differs from that of the analysis dimension: the analysis dimension reached its peak and stabilized by the third session, whereas the support dimension did not achieve significant improvement until the fourth session. This temporal discrepancy may reflect differences in the developmental logic of the two competencies. Analytical ability relies more heavily on the establishment of cognitive schemas, which, once formed, tend to remain relatively stable. In contrast, support ability requires, on the basis of analysis, the further integration of information about young children’s backgrounds, educational goals, and practical feasibility, representing a higher-order comprehensive competency whose development necessitates a longer accumulation process.

In summary, during the sequential exposure to diverse AV, the development of TCs’ observational skills follows a trajectory characterized by differentiated changes across dimensions: the observation dimension becomes largely established in the initial stage of skill acquisition and remains stable in subsequent training; the analysis dimension shows significant progress initially, then enters a phase of consolidation, achieving a notable improvement after the third workshop; the support dimension exhibits a continuous upward trend, with significant improvement observed after the fourth workshop. This developmental trajectory suggests that the sequential integration model may support the progressive maturation of higher‑order cognitive abilities through staged practice. However, these patterns should be interpreted as preliminary given the exploratory nature of the study and the absence of a control group. Further research with controlled designs is needed to confirm these developmental patterns.

#### 4.2.2. Longitudinal changes in self-assessed observation knowledge.

To examine the changes in students’ subjective perceptions of their mastery of observational knowledge during sequential exposure to AV, this study conducted paired-sample t-tests on the scores from the “observational knowledge” dimension of the questionnaires completed by 34 core sample students at three time points. The three time points were: T1 (after the VS), T2 (after the first AV, corresponding to the theme of independent reading conflicts in the intermediate class), and T3 (after the completion of all four workshops). Since the “observational knowledge” dimension comprises disciplinary knowledge (items 6–8) and methodological knowledge (items 9–14), this study calculated the changes in disciplinary knowledge, methodological knowledge, and the total score across the three time points, with the results presented in Table 5. Given the small sample size, non‑parametric Friedman tests were also conducted as robustness checks, and the results were consistent with the parametric tests. Effect sizes (Cohen’s d) are reported for significant comparisons.

**Table 5:** Longitudinal trends in self-rated observational knowledge.

	M	SD	Paired-Samples t-Test (t/p)	Cohen’s d	95% CI		Non-parametric Method (Wilcoxon Signed-Rank Test)
					Lower Bound	Upper Bound	
**XK1**	3.80	0.51	XK1 - ZK1, −1.890(0.068) XK1 - TK1, −1.927(0.063) ZK1 - TK1, −0.220(0.827)	XK1 - ZK1,-0.324	−0.667	0.023	−1.915(0.055)
**ZK1**	3.97	0.48		XK1 - TK1,-0.330	−0.673	0.017	−2.054*(0.040)
**TK1**	3.99	0.57		ZK1 - TK1, −0.038	−0.374	0.299	−0.180(0.857)
**XK2**	3.76	0.66	XK2 - ZK2, −2.492*(0.018) XK2 - TK2, −2.204*(0.035) ZK2 - TK2, −0.355(0.725)	XK2 - ZK2,-0.427	−0.776	−0.073	−2.459*(0.014)
**ZK2**	4.01	0.47		XK2 - TK2,-0.378	−0.723	−0.027	−2.443*(0.015)
**TK2**	4.04	0.52		ZK2 - TK2,-0.061	−0.397	0.276	−0.617(0.537)
**XK**	3.77	0.55	XK – ZK, −2.550*(0.016) XK – TK,-2.247*(0.031) ZK – TK, −0.340(0.736)	XK – ZK,-0.437	−0.787	−0.082	−2.284*(0.022)
**ZK**	4.00	0.42		XK – TK,-0.385	−0.731	−0.034	−2.489*(0.013)
**TK**	4.02	0.50		ZK – TK,-0.058	−0.394	0.278	−0.586(0.558)

Note: *p < 0.05.

Regarding the dimension of disciplinary knowledge (reflecting students’ understanding and recognition of early childhood education theories), scores exhibited a gradual upward trend: T1 was 3.80 ± 0.51, T2 was 3.97 ± 0.48, and T3 was 3.99 ± 0.57. However, paired-sample t-tests revealed that none of the pairwise comparisons between the three time points reached statistical significance (T1–T2: p = 0.068; T1–T3: p = 0.063; T2–T3: p = 0.827). This finding suggests that students’ self-perceived improvement in theoretical knowledge during the sequential training process was relatively limited, potentially indicating that the accumulation of disciplinary knowledge requires a longer duration of training or more theoretically in-depth learning activities.

In the dimension of methodological knowledge (reflecting students’ perceived mastery of teaching strategies, interactive skills, practical application, etc.), scores exhibited a significant increase followed by a stabilization trend. Specifically, the score at T1 was 3.76 ± 0.66, which significantly increased to 4.01 ± 0.47 at T2 (t = −2.492, p = 0.018 < 0.05, Cohen’s d = [−0.427], 95% CI for the mean difference [−0.776, −0.073]. The non-parametric Wilcoxon signed-rank test yielded consistent results (z = −2.459, p = 0.014 < 0.05), indicating that the findings for this dimension are statistically robust. The score at T3 was 4.04 ± 0.52, showing no significant difference compared to T2 (t = −0.355, p = 0.725 > 0.05), but remaining significantly higher than that at T1 (t = −2.204, p = 0.035 < 0.05, Cohen’s d = [−0.378], 95% CI for the mean difference [−0.723, −0.027]. The non-parametric Wilcoxon signed-rank test yielded consistent results (z = −2.443, p = 0.015 < 0.05), indicating that the findings for this dimension are statistically robust. This trajectory of rapid initial progress followed by steady consolidation suggests that students’ perceived methodological knowledge achieved a qualitative breakthrough after their first exposure to AV, and was subsequently reinforced through continued training.

The trend in the total score for observation knowledge closely mirrored that of methodological knowledge. The total score at T1 was 3.77 ± 0.55, which significantly increased to 4.00 ± 0.42 at T2 (t = −2.550, p = 0.016 < 0.05, Cohen’s d = [−0.437], 95% CI for the mean difference [−0.787, −0.082]. The non-parametric Wilcoxon signed-rank test yielded consistent results (z = −2.284, p = 0.022 < 0.05), indicating that the findings for this dimension are statistically robust. And reached 4.02 ± 0.50 at T3, showing no significant difference from T2 (t = −0.340, p = 0.736 > 0.05) but remaining significantly higher than T1 (t = −2.247, p = 0.031 < 0.05, Cohen’s d = [−0.385], 95% CI for the mean difference [−0.731, −0.034]. The non-parametric Wilcoxon signed-rank test yielded consistent results (z = −2.489, p = 0.013 < 0.05), indicating that the findings for this dimension are statistically robust. This finding suggests that the growth in students’ self-perceived observation knowledge was primarily concentrated in the initial stage of transitioning from VS to AV, with subsequent training mainly serving a consolidating role.

Based on the above analysis, students’ self-assessment of observational knowledge during sequential exposure to AV demonstrates dimension-specific developmental characteristics. Disciplinary knowledge showed slow improvement and did not reach a significant level, potentially reflecting that the deep internalization of theoretical knowledge requires a longer period of learning accumulation. Methodological knowledge exhibited a significant increase after the first exposure to AV and subsequently remained stable, indicating that the perception of practical knowledge is more readily acquired through exposure to real-world contexts. The overall score for observational knowledge followed a trajectory consistent with that of methodological knowledge, characterized by rapid initial progress followed by stable consolidation, suggesting that the growth in students’ overall self-perception was primarily driven by the enhancement of methodological knowledge.

It should be noted that the results reported above reflect students’ subjective perceptions of their observational knowledge mastery, rather than objective performance measures. The questionnaire data and the group observation assignment scores capture training effects from two distinct dimensions-subjective experience and objective outcomes-and should be viewed as complementary rather than equivalent indicators. Self-reported knowledge gains may be influenced by factors such as changes in confidence, social desirability effects, and understanding of scoring criteria. Therefore, these findings should be interpreted with caution and should not be equated with actual competence improvement.

#### 4.2.3. Comparative analysis of subjective perception and objective performance.

To further understand the developmental characteristics of students during sequential training, this study conducted a comparative analysis between students’ self-reported trajectories of “observational knowledge” and the objective scores (analytical dimension) of their group assignments. It should be noted that, due to the research design, group assignments were collected after each workshop, whereas the questionnaire survey covered three time points: the first workshop, the second workshop, and the end of the entire intervention. Therefore, this section focuses on comparing the trends of change between the first and second workshops to examine the consistency between subjective perceptions and objective performance. Given the differences in measurement scales (the subjective self-report used a 5-point scale, while the objective score ranged from 0 to 30), this section emphasizes the directionality, significance, and stage-specific differences of the changes, rather than a direct comparison of absolute values.

The total score for students’ subjective perception of observational knowledge increased from 4.00 to 4.02, representing an improvement of 0.02 points (p = 0.735 > 0.05), which was not statistically significant. Over the same period, the score for the objective analysis dimension (Table 4) rose from 24.75 to 25.84, an increase of 1.09 points (p < 0.001), indicating a significant improvement. In terms of the direction of change, both the total score for subjective perception of observational knowledge and the score for the objective analysis dimension exhibited an upward trend, with neither showing a decline, suggesting that students maintained or enhanced their observational skills during the later stage of training. Regarding the stage at which significant changes occurred, a clear temporal sequence emerged: the significant improvement in subjective perception took place at an earlier stage, whereas the significant increase in objective performance occurred at a slightly later stage. This temporal order may reveal differences in the developmental logic underlying the two dimensions. Specifically, after being exposed to authentic contexts, students first experienced an increase in their perceived competence at the subjective level; this positive shift in self-perception may subsequently serve as motivation for continued engagement in learning, thereby promoting actual improvement in objective performance. In other words, the enhancement of subjective perception may act as a psychological precursor or early signal for the subsequent leap in objective competence. This finding aligns with metacognitive development theory, which posits that improvements in metacognitive ability often precede changes in actual performance, and that positive changes in self-perception may serve as a driving force for sustained engagement in learning.

### 4.3. Learners’ perceptions and evaluation of the sequential integration model

To address the third research question, this section presents a thematic analysis of learners’ subjective experiences and perceived value regarding the “VS–AV” sequential integration model, based on in-depth interviews with 9 students. Through systematic analysis, three core themes were identified: the challenge of finding meaning in chaos, the desire for structured support, and the awareness of one’s own growth. Each of these themes is elaborated below. The following qualitative findings represent participants’ subjective perceptions and experiences. They are presented not as direct evidence of actual skill improvement, but as complementary evidence that helps interpret and contextualize the quantitative patterns observed.

#### 4.3.1 The challenge of finding meaning in chaos.

First, difficulties in identification and judgment were reported by students. During the observation process, the primary challenge perceived by students was the difficulty in accurately identifying the subject of observation and interpreting young children’s behavioral intentions and emotional states. In the VS, this difficulty stemmed mainly from the crude presentation and lack of information. Student S2 noted, “I feel that those virtual videos are sometimes rough. Sometimes the text does not match the images in the video, the subtitles are inconsistent, and sometimes you simply cannot see anything in the video, so you have to rely on the subtitles to analyze.” In contrast, in AV, the subject of observation was ambiguous, and the large number of young children with messy behaviors made it difficult for students to pinpoint the core target of observation or determine the nature of the behavior. Student S2 further stated, “In real-life situations, there is no teacher explaining the subject in advance or providing a brief summary of what is happening. Sometimes, just by watching the video, it is unclear who exactly is the main subject of observation.”

Secondly, difficulties in information processing. During the observation process, students commonly faced issues such as disorganized information, missing key details, or unclear presentation, which made it difficult to effectively process and analyze the observed information. In AV, the information was often disorganized, with numerous irrelevant distracting factors, requiring students to expend considerable effort to filter out the effective information. Student S8 admitted: “In AV, you have to summarize on your own; it’s not as simple and clear as the virtual videos. You might need to watch them several times to figure out the logic. The videos cover multiple themes, which require careful identification, and sometimes mistakes can happen.” In virtual scenarios, difficulties in information processing mainly stemmed from the irregular presentation and thin content of the videos. Student S2 stated: “Some virtual videos are quite rough, with mismatched text and images, inconsistent subtitles. Sometimes it’s hard to discern anything from the video itself, and you have to rely on the subtitles to analyze.”

Thirdly, difficulties in process management. During the observation workshops, students also faced challenges such as unreasonable observation procedures and a lack of effective guidance, making it difficult to carry out observation activities in an orderly manner. On the one hand, the workshops were held intensively with heavy workloads, which hindered students’ ability to absorb the material. Student S9 said: “The four workshops were too intensive [...] This time, we learned all the methods first and then did the workshops. Points I didn’t pay attention to at the beginning could only be patched up later.” On the other hand, students lacked practical experience in observation methods, making it difficult to flexibly choose appropriate methods for different observation scenarios. Student S8 noted: “The biggest challenge with AV was sorting out the logic. It was difficult at first, but once I got things sorted out, the subsequent analysis went more smoothly.”

#### 4.3.2 Aspirations for structured support.

First, there was a demand for standardized support. Students generally expressed a need for standardized guidance and preparatory support for observation, aiming to clarify the focus, procedures, and methods, thereby reducing uncertainty during the observation process. Prior to observation, students required clear prompts highlighting the focus of the observation, along with preparatory materials. Student S3 suggested: “Before the observation, we could be given clearer instructions.” Student S7 also noted: “Could the materials be sent out a few days in advance so that we have time to choose the observation methods we want to use?” During observation, students needed standardized observation tools and process guidance. Student S7 further stated: “For certain observation materials and videos, we could be provided with other behavior checklists or similar tools. We could prepare checklists or charts in advance and think about whether to use them, rather than only relying on continuous real-time recording.”

Second, there was a need for presentation optimization. Students articulated specific demands for improving the presentation quality of both virtual and authentic observation videos, hoping that enhancements to video presentation would compensate for information gaps and reduce difficulties in identification and information processing. Regarding virtual videos, students sought greater precision in video production and richer content presentation. Student S1 stated: “The virtual content could be slightly richer, with more presentation of children’s behaviors and speech, to make the content more substantial.” Regarding AV, Student S7 noted: “The footage of AV can be shaky, making the presentation somewhat fragmented [...] Additionally, I hope supplementary background information could be provided, such as the children’s age group and the context of the event, to avoid abruptness that leaves us without a starting point.”

Third, there was a demand for improved efficiency. Students hoped to enhance the efficiency of observation and analysis by streamlining the observation process and providing targeted guidance, thereby reducing time-consuming and labor-intensive issues. For instance, regarding methodological guidance, students expressed a need for practical instruction on observation methods. Student S8 stated: “After each analysis, the teacher could, from a professional perspective, help us analyze the children’s behaviors further, integrating relevant theories to explain the reasons behind those behaviors. This would help us master observation and analysis methods more quickly and improve efficiency.”

#### 4.3.3 Awareness of one’s own growth.

First, professional competence was enhanced. By participating in the observation activities, students’ observational skills, adaptability, and professional practical abilities were significantly improved. For example, with regard to observational skills, Student S8 stated, “Watching AV allows me to observe better and in more detail. At the beginning, I did not pay attention to small details, but later, influenced by my group peers, I started to notice more details, and my observational skills improved.” Regarding adaptability, Student S5 noted, “Observing authentic contexts helps improve our adaptability. In authentic contexts, children’s thoughts are changeable and their behaviors are flexible, so teachers need to respond flexibly. This reminds me that as a future preschool teacher, I need to improve my adaptability and learn to flexibly respond to children’s various behaviors.”

Second, cognitive understanding was deepened. The observation activities gave students a deeper awareness and understanding of authentic kindergarten teaching scenarios, characteristics of children’s behaviors, the role of teachers, and the relationship between theory and practice. In terms of understanding children’s behaviors and authentic teaching scenarios, Student S8 said, “Through AV, I can gain a more intuitive understanding of frontline teaching scenarios and see children’s authentic reactions more clearly.” Regarding the perception of the teacher’s role, Student S1 stated, “When watching AI-based VS videos, I tended to focus more on the teacher’s role-how the teacher responded to children’s behaviors. When watching AV, I paid more attention to the children’s performance and feedback. Through this comparison, I gained a clearer understanding of the teacher’s role as a guide and responder in children’s activities.” With respect to the relationship between theory and practice, Student S6 admitted, “Through observation and kindergarten practicum experiences, I realized that there is a gap between the knowledge I learned and the actual work, and I gained a deeper understanding of the importance of integrating theory and practice.” It is worth noting that S6’s reference to practicum experiences drew on her prior fieldwork completed before the intervention, rather than experiences gained during the workshops themselves. This awareness of the “limitations of theory” is a crucial indicator of deepened professional knowledge.

Third, accumulation of practical experience was fostered. By participating in the observation activities, students accumulated substantial practical experience related to observing children’s behaviors and teachers’ response strategies, providing strong support for their future internships and professional development. On the one hand, the observation activities familiarized students in advance with the authentic work scenarios in kindergartens and the challenges they might face. Student S2 noted, “Observation in authentic contexts is crucial, as it prepares you for future work and internships.” On the other hand, through hands-on observation, students gained practical experience in conducting observational records and behavioral analyses. Student S7 mentioned, “Through the workshop, I understood the knowledge structure of observation, learned how to do observational records and think about support strategies, and accumulated experience in analyzing children’s behaviors.”

Synthesizing the findings from the three core themes above, learners’ perceptions of the sequential integration model combining VS and AV reveal a complete cognitive trajectory, moving from “challenge” to “demand” and then to “growth”. In the initial phase, students experienced cognitive conflicts and adaptation difficulties when transitioning from virtual to authentic contexts. In the intermediate phase, students expressed diverse needs for structured support. In the later phase, students achieved positive growth in professional competence, cognitive understanding, and practical experience. This trajectory illustrates how learners experienced the sequential integration model—from initial challenges, through expressed needs for support, to perceived professional growth. These findings suggest that the model may help students not only acquire observational skills but also develop metacognitive awareness of their own professional growth, which may support their ongoing professional development.

## 5. Discussion

This study aims to explore the sequential integration pathway of VS and AV in observational skills training for TCs. Through a DBR approach, it systematically examines the differential effects of the two media in the initial stages of skill acquisition, the evolution of observational skills during the sequential engagement with AV, and learners’ perceptions and value judgments of the integrated model across three dimensions: objective performance, subjective self-assessment, and qualitative experience. The following discussion is structured around three research questions.

### 5.1. The complementary roles of VS and AV in the initial stages of skill acquisition

The first core finding of this study is that, in the initial stages of skill acquisition, VS and AV exert differential effects on the observational performance and observation quality of preservice preschool teachers, exhibiting complementary characteristics. However, as noted in the Results section, the comparison between Workshop 1 (VS) and Workshop 2 (AV) was subject to confounding factors—including differences in scenario content, workshop order, and the additional teacher sharing session in Workshop 2. Therefore, the observed differences cannot be solely attributed to media type, and the following interpretations should be considered tentative.

At the level of objective performance, AV showed higher scores than VS in both the analysis dimension and the support dimension. This finding diverges from some existing research. Previous studies have often emphasized the advantages of virtual simulation in reducing cognitive load and providing a “safe practice environment” for beginners [15,31]. However, this study found that although the virtual simulation context was more simplified, students demonstrated higher analytical performance under the AV condition. This may stem from the fact that the contextual complexity presented by AV constitutes cognitive material “worthy of analysis”. As distinguished by CLT, extraneous cognitive load (caused by poor instructional design) differs in nature from germane cognitive load (resulting from learners’ active schema construction) [24]. Although AV increases extraneous cognitive load, its rich behavioral details and interactive cues may also provoke higher germane cognitive load, prompting students to invest more cognitive resources in deep processing. This finding suggests that in the initial stages of skill acquisition, learning contexts should not be oversimplified; moderate complexity may, in fact, act as a catalyst for deep cognitive processing [32]. Nevertheless, this interpretation remains tentative. The observed advantages of AV in analysis and support dimensions may also reflect the effects of practice, the additional expert sharing session, or the different scenario content, rather than media type alone. Future studies employing controlled experimental designs are needed to isolate the unique contribution of each medium.

At the level of subjective observation quality, VS significantly enhanced students’ purposefulness (active thinking), while AV significantly enhanced their sensitivity (detail capture). This finding echoes the research by Badilla-Quintana and Sandoval-Henríquez on “interactivity” and “sense of presence” in immersive experiences [33]. The clarity and controllability of the virtual simulation environment provide students with space for “focused reflection,” allowing them to concentrate their attention on thinking about teaching events. Conversely, the complexity and authenticity of the AV environment compel students to maintain a high level of alertness to capture subtle changes in young children’s behavior [34,35]. The complementarity of the two media in cultivating observational qualities provides a theoretical basis for sequential integration design. It should be noted, however, that the robustness of this finding was insufficient (Wilcoxon signed-rank test, p = 0.053), suggesting caution in interpretation.

Qualitative materials further corroborate these findings. Students commonly reported that the VS was “well-structured” and “quite intuitive”, facilitating reflection, whereas the AV allowed them to “observe more meticulously” and “notice more details”. Notably, students’ descriptions of the AV as “information-rich” and “requiring one’s own synthesis” precisely reflect their active construction process under high cognitive load.

Taken together, this finding offers a nuanced perspective on the assumption that “virtual simulation, as an initial scaffold, is necessarily superior to AV,” revealing the functional complementarity of the two media in the initial stages of skill acquisition. This finding offers important insights for subsequent sequential integration design: the value of VS lies not in “replacing” AV, but in providing learners with a cognitive space conducive to reflective thinking; the value of AV lies in the natural impetus for cognitive processing provided by its contextual authenticity.

### 5.2. Sequential integration pathway: Dimensionally differentiated trajectories of observational skills development

The second core finding of this study is that, during the process of sequentially engaging with a diverse range of AV ranging from typical to complex, TCs’ observational skills exhibited dimensionally differentiated developmental trajectories.

In the analysis dimension, students’ competence progressed through stages of “significant leap-consolidation plateau”: the analysis dimension score was 24.75 after the second workshop, increased significantly to 25.84 after the third, and remained stable after the fourth. However, caution is warranted when interpreting the improvement from the second to the fourth workshop. Although the paired-samples t-test indicated a significant improvement (p = 0.038), the non-parametric Wilcoxon test did not reach statistical significance (p = 0.057), suggesting that this difference lacked sufficient statistical robustness. This pattern—a significant leap followed by a plateau—therefore primarily reflects a genuine breakthrough between the second and third workshops, rather than a sustained linear progression across all three time points. This “leap-plateau” trajectory aligns with the “rapid progress period-consolidation period” model in skill development theory [36,37]. Notably, this leap occurred precisely between the second and third workshops, coinciding with a thematic shift from “conflict during self-directed reading in a middle kindergarten class” to “separation anxiety in a junior kindergarten class”. This change in topic may have introduced new analytical challenges, allowing students to achieve a breakthrough in competence as they addressed these challenges. Furthermore, inviting a practicing kindergarten teacher to share their experiences after the second workshop may have provided students with a crucial learning scaffold.

In the support dimension, students’ competence exhibited a continuous upward trend, achieving a significant increase only after the fourth workshop. This finding echoes Kolb’s experiential learning theory [38]. Support competence (formulating educational recommendations) requires higher-order integrative abilities compared to analysis competence (interpreting behavior). It demands not only understanding the reasons for young children’s behavior but also synthesizing information about the child’s background, educational goals, and practical feasibility to formulate actionable intervention plans. The cultivation of this competence requires a longer accumulation period [39], which is consistent with the finding of delayed development in the support dimension observed in this study.

In the observation dimension, students’ competence largely stabilized in the initial stages of skill acquisition and remained steady during subsequent training. This finding may reflect the characteristic of the observation dimension as a foundational competence: after students acquire basic observational description skills, subsequent training primarily serves to consolidate and refine these skills rather than produce quantitative leaps.

It is noteworthy that the developmental trajectory of subjectively perceived observational knowledge exhibited a temporal lag relative to objective performance: the period in which students perceived significant improvement did not coincide with the period in which objective performance showed significant gains. This finding reveals the asynchronicity between self-awareness and actual competence development. After being exposed to authentic contexts, students first experienced subjectively that “they seemed to understand more”, and only later did their actual performance demonstrate a competence leap. This finding is consistent with metacognitive development theory: improvements in metacognitive ability often precede changes in actual performance [40], and positive changes in self-perception may serve as motivation for sustained learning engagement.

Taken together, the value of the sequential integration model lies not only in promoting the overall improvement of observational skills but also in its differentiated effects on the cultivation of distinct skill dimensions. This finding provides empirical evidence for teacher education curriculum design: training designs need to account for the developmental rhythms of different skill dimensions, providing timely challenges during the “leap period” for analytical competence, and offering sustained practice opportunities during the “accumulation period” for support competence.

A key question for teacher education is whether observational skills developed through video-based training transfer to authentic classroom practice. While this study did not directly assess transfer to real teaching, students’ qualitative reports suggested perceived transfer—several participants mentioned feeling better prepared for kindergarten practicum. However, the extent to which these perceptions correspond to actual transfer remains an open question. Future research should include direct measures of transfer, such as classroom observations or performance assessments in authentic settings, to examine the generalizability of the skills developed through sequential training.

### 5.3. Learners’ experiential trajectory: From cognitive conflict to metacognitive growth

The third core finding of this study is that learners’ experiences with the “VS-AV” sequential integration model exhibited a complete trajectory, progressing from “cognitive conflict” to “support needs”, and ultimately to learning to reflect on your own thinking and understand how you learn.

In the initial stage, students encountered cognitive conflict and adaptation difficulties during the transition from virtual to authentic contexts. Difficulties in recognition and judgment, information processing, and procedural navigation constituted the “challenge of finding meaning in chaos”. This finding resonates with the transitional characteristics of “a process where beginners learn by taking part in simple, less central tasks within a community, gradually moving toward full participation as they gain experience” in SLT [25,41]. As students moved from “simplified training contexts” to “authentic practical contexts”, they inevitably experienced cognitive conflict and adaptation difficulties. While this process may cause discomfort, it represents an essential phase of learning.

In the intermediate stage, students expressed diverse needs for structured support, including the need for standardization (clear guidance, checklists), presentation optimization (video quality, contextual background), and efficiency enhancement (methodological guidance). These needs essentially point to a core demand: during the transition from Virtual Simulation to AV, students require more refined scaffolding support [42,43]. This finding offers direct implications for the design of sequential training models: the sequential arrangement of media types constitutes only one aspect of an integrated model; supporting tools, procedural frameworks, and feedback mechanisms are equally critical.

The qualitative patterns reported here align with the quantitative findings presented in Sections 4.1 and 4.2. For instance, students’ perception of AV as more cognitively demanding corresponds to the higher analytical scores but lower purposefulness self-ratings observed in the AV condition. This convergence suggests that the cognitive challenge posed by authentic videos may simultaneously enhance analytical performance while reducing students’ perceived capacity for reflective thinking—a trade-off that the sequential integration model was designed to manage.

In the later stage, students achieved positive growth in professional competence, cognitive understanding, and practical experience. Notably, students’ awareness of their own growth was evident not only in skill-level improvements such as “enhanced observation skills”, but also in metacognitive dimensions such as “deepened pedagogical understanding” and “strengthened professional confidence” [44,45]. Student S6’s profound recognition of the “gap between theory and practice”, as well as Student S1’s contrast in role perception between “VS video focusing on the teacher, AV focusing on the child”, both reflect the reflexive awareness of their own learning processes that students developed through the sequential training [46].

This finding offers preliminary support for the value of the sequential integration model in enhancing teacher professional competence. The “awareness of one’s own growth process” that students reported in this study may constitute a metacognitive foundation for ongoing professional development in teachers. However, given the exploratory nature and the absence of a control group, this interpretation should be confirmed in future research.

## 6. Conclusions

### 6.1. Conclusion

Through a design-based intervention approach, this study systematically examined the sequential integration pathway of VS and AV in observational skills training for preschool TCs. The findings reveal that, in the initial stages of skill acquisition, VS is more conducive to stimulating students’ active thinking, whereas AV is more effective in refining their ability to capture details, with the two media exhibiting functional complementarity. During the sequential engagement with AV, analytical competence followed a “leap-plateau” trajectory, support competence demonstrated a continuous upward trend, and observation competence remained stable. Learners’ experiences followed a complete trajectory progressing from “cognitive conflict” to “support needs” and ultimately to “metacognitive growth”. These findings offer preliminary support for the value of the sequential integration model in promoting professional development in teachers, providing both theoretical foundations and practical implications for teacher education curriculum design. The “VS—AV” sequential integration model examined in this study may offer a useful framework for cultivating high-quality preschool education teachers. However, these conclusions should be interpreted in light of the study’s exploratory nature and the limitations discussed below.

### 6.2. Limitations

Sample limitations. This study selected only one intact class of students as the research subjects, resulting in a relatively small sample size (N = 46) and a low proportion of male students (8 individuals). Although the study employed multi-round data collection and a mixed-methods design to enhance the robustness of the conclusions, the generalizability of the findings still requires validation with larger and more diverse samples. Future research could expand the sample scope to examine the influence of factors such as gender and learning style on the effects of sequential training.

Study duration limitations. The study was conducted over a period of six weeks. While it captured the preliminary trajectory of observational skills development, it did not examine the long-term retention or transfer of training effects. The lasting impact and transferability of the competencies developed through this training therefore remain unknown. Future research could design longitudinal studies to investigate the impact of sequential training on students’ actual teaching performance after graduation.

Control limitations in research design. This study employed a single-group design and did not include a control group to verify the superiority of the sequential integration model over training using a single medium. Future research could adopt quasi-experimental designs to compare the effects of different models, such as “VS first followed by AV” “AV first followed by VS” or “AV only” in order to further clarify the unique value of sequential integration.

These limitations—particularly the single-group design, small sample, and lack of control group—mean that the findings should be interpreted as exploratory rather than confirmatory. The sequential integration model shows promise, but its effectiveness and generalizability remain to be established through more rigorous experimental designs.

### 6.3. Implications

At the theoretical level, this study makes three contributions. First, it reveals the functional complementarity of VS and AV in the initial stages of skill acquisition—VS provides students with a cognitive space conducive to active thinking through contextual simplification, while AV stimulates deep cognitive processing through contextual complexity. This finding concretizes CLT into a differentiated basis for media selection in teacher training. Second, it proposes a “dimensionally differentiated” model of observational skills development, revealing that analytical competence progresses through a “leap-plateau” trajectory, support competence exhibits continuous upward growth, and observation competence largely stabilizes in the initial stage—thereby challenging traditional conceptions that treat observational skills as a unidimensional competence. Third, it constructs a three-stage trajectory of learner experience—“cognitive conflict-support needs—metacognitive growth”—revealing the psychological mechanism underlying the transition from “guided observer” to “reflective practitioner” and offering a new theoretical explanation for understanding teacher professional development.

At the practical level, this study provides four implications. First, it offers a “VS-first-then-AV” sequential design framework for technology integration, emphasizing a cognitive trajectory from “reflection” to “practice”. Second, it proposes differentiated support strategies, providing challenging tasks during the “leap period” for analytical competence, sustained practice opportunities during the “accumulation period” for support competence, while responding to students’ needs for structured support such as checklists and clear guidelines. Third, it provides a pathway for cultivating metacognition in teacher professional development, guiding students to develop awareness of theoretical limitations and their own growth through reflective writing and similar approaches. Fourth, it offers specific standards for video resource development—VS should avoid oversimplification, while AV should prioritize recording quality and be supplemented with contextual background information to reduce ineffective cognitive load.

## Supporting information

S1 Data Student Questionnaire Data. Raw data from the student questionnaire on self-perceived observation knowledge, collected at three time points (T1, T2, T3). The file includes subscale scores for purposefulness, organization, comprehensibility, accuracy, acuity, persistence, originality, theory application, and method application, as well as total scores.
(CSV)

S1 Table Scoring Rubric for Group Observation Assignments. Detailed scoring criteria for evaluating group analytical texts across three dimensions: Observation (40 points), Analysis (30 points), and Support (30 points). The table provides performance levels (Excellent to Needs Improvement) with specific descriptors and score ranges for each level.
(DOCX)

S1 Text Interview Coding Scheme and Excerpts. Coding framework derived from reflexive thematic analysis of semi-structured interviews with nine participants. The framework comprises 3 core themes, 9 cluster codes, and 26 open codes. Representative verbatim excerpts are provided to illustrate each theme.
(DOCX)

S2 Data Group Observation Scores (CSV). Scores of eight groups on the Observation, Analysis, Support, and Total dimensions for each of the four workshops. Missing values (NA) for Workshop 3 Observation and Support are indicated, as only the Analysis dimension was assessed in that round.
(CSV)

S2 Table Workshop Protocol. Summary of the four sequential workshops, including video types (virtual simulation vs. authentic video), themes/scenarios, student activities, data collection points, and instructor/expert support. Additional notes on video production and potential confounding factors are included.
(DOCX)
