# Supplementary material for: A sequential integrating virtual simulation and authentic video for preschool teacher candidates’ observational skills training: A design-based intervention study
Source: PLoS One. 2026 Sep 21;21(9):e0358951. doi: 10.1371/journal.pone.0358951 (PMC13592612; doi:10.1371/journal.pone.0358951)
Supplement: Supplementary file 1 [file pone.0358951.s002.docx]

**S1 Table. Scoring Rubric for Group Observation Assignments (DOCX)**

**-Scoring Rubric S1 Table**

**S1 Table . Scoring Rubric for Group Observation Assignments**

| **Scoring Dimension** | **Total Score** | **Rating Level** | **Score Range** | **Level Description** |
| --- | --- | --- | --- | --- |
| **Observation Dimension**Assesses:Objectivity of behavioral description,Completeness of contextual information,Accuracy of detail capture | 40 points | Excellent | 36–40 points | 1. Behavioral descriptions are fully objective and factual, presenting only overt children's actions, language, and expressions without any subjective evaluation or inferential statements. |
|  |  |  |  | 2. Comprehensively covers three core categories of contextual information: physical environment, peer interaction processes, and teacher intervention approaches. |
|  |  |  |  | 3. Precisely captures subtle details such as micro-expressions and minor movements; the event timeline is complete and clear, enabling full reconstruction of the entire behavioral process. |
|  |  | Good | 32–35 points | 1. Behavioral descriptions are generally objective with clear core facts; only very few expressions carry mild subjective tendencies that do not affect overall judgment. |
|  |  |  |  | 2. Presents two or more categories of contextual information with no omission of key behavioral nodes. |
|  |  |  |  | 3. Behavioral details are relatively rich, the event sequence is coherent, and the complete behavioral process can be largely reconstructed. |
|  |  | Satisfactory | 24–31 points | 1. Core behaviors are described, with a small amount of subjective evaluation interspersed; some secondary facts are vaguely expressed. |
|  |  |  |  | 2. Only mentions 1–2 categories of contextual information with incomplete content. |
|  |  |  |  | 3. The event process is generally traceable, with minor omissions at key nodes. |
|  |  | Acceptable | 16–23 points | 1. Core behaviors are identifiable, but accompanied by considerable subjective inferences and vague factual expressions. |
|  |  |  |  | 2. Only scattered references to a single contextual factor with incomplete information. |
|  |  |  |  | 3. Event descriptions are not sufficiently coherent, with obvious omissions of key behavioral nodes. |
|  |  | Needs Improvement | 0–15 points | 1. Subjective evaluation replaces objective behavioral description, resulting in significant deviations from behavioral facts. |
|  |  |  |  | 2. Barely mentions relevant contextual factors, which cannot support subsequent analysis. |
|  |  |  |  | 3. Event logic is disordered, making it impossible to reconstruct the basic process of the behavior. |
| **Analysis Dimension**Assesses: Theory alignment,Depth of interpretation,Logical rigor,Correspondence with observed facts | 30 points | Excellent | 26–30 points | 1. Precisely applies professional theories from child development psychology and early childhood education to interpret behaviors, with accurate and standardized theoretical references. |
|  |  |  |  | 2. Analytical logic progresses incrementally, providing in-depth explanations of the developmental mechanisms, internal needs, and educational significance underlying behaviors. |
|  |  |  |  | 3. Analytical content highly corresponds to observed behavioral facts, achieving "reasoning grounded in behavior" with no disconnection between theory and facts. |
|  |  | Good | 24–25 points | 1. Correctly applies relevant professional theories to interpret behaviors with no obvious errors in theoretical references. |
|  |  |  |  | 2. Possesses clear analytical logic, able to illustrate the developmental level and typical characteristics corresponding to the behavior. |
|  |  |  |  | 3. Analytical content corresponds to observed facts with good relevance, though in-depth elaboration is slightly insufficient. |
|  |  | Satisfactory | 18–23 points | 1. References relevant theories with generally accurate expressions, but the connection to behaviors remains relatively superficial. |
|  |  |  |  | 2. Provides basic attribution of behaviors with generally coherent analytical logic. |
|  |  |  |  | 3. Analytical content has correspondence with observed facts but is not sufficiently close. |
|  |  | Acceptable | 12–17 points | 1. Theoretical expressions are not sufficiently accurate, and the connection to behaviors is relatively far-fetched. |
|  |  |  |  | 2. Only provides simple attribution, with insufficient analytical depth and unclear logic. |
|  |  |  |  | 3. The correspondence between analytical content and observed facts is weak, with partial disconnection. |
|  |  | Needs Improvement | 0–11 points | 1. Fails to cite relevant professional theories, or theoretical references contain obvious common-sense errors. |
|  |  |  |  | 2. Only restates observed behavioral facts without substantive interpretation or analysis. |
|  |  |  |  | 3. Analytical logic is disordered, with no clear connection to observed behaviors. |
| **Support Dimension**Assesses: Needs alignment, Operability,Diversity of strategies, Context adaptability | 30 points | Excellent | 27–30 points | 1. Support strategies closely echo children's developmental needs derived from analysis, forming a complete logical closed loop with the preceding analysis. |
|  |  |  |  | 2. Educational suggestions are specific and operable, covering multiple pathways including environment creation, teacher-child interaction, play support, and home-kindergarten collaboration. |
|  |  |  |  | 3. Fully integrates children's age characteristics, prior experience, and scenario background, with strong practical applicability. |
|  |  | Good | 24–26 points | 1. Support strategies generally match children's developmental needs with clear correspondence to analytical content. |
|  |  |  |  | 2. Proposes two or more specific educational suggestions with basic operability. |
|  |  |  |  | 3. Takes into account children's age-related developmental characteristics, conforming to the reality of kindergarten educational scenarios. |
|  |  | Satisfactory | 18–23 points | 1. Support strategies have certain relevance to analytical conclusions with average targeting. |
|  |  |  |  | 2. Proposes general educational directions, but specific implementation pathways are not clear enough. |
|  |  |  |  | 3. Generally conforms to children's age characteristics with acceptable context adaptability. |
|  |  | Acceptable | 12–17 points | 1. The relevance between support strategies and analytical conclusions is weak, with insufficient targeting. |
|  |  |  |  | 2. Suggestions are relatively vague and general, with poor operability. |
|  |  |  |  | 3. Rarely integrates children's specific characteristics, being broadly applicable but insufficiently adapted to the scenario. |
|  |  | Needs Improvement | 0–11 points | 1. Support strategies are disconnected from preceding analytical content and fail to address children's developmental needs. |
|  |  |  |  | 2. Suggestions are empty and abstract, with no specific implementation pathways and no practical operability. |
|  |  |  |  | 3. Inconsistent with children's age-related developmental patterns and the reality of kindergarten educational scenarios. |
