# Supplementary material for: A sequential integrating virtual simulation and authentic video for preschool teacher candidates’ observational skills training: A design-based intervention study
Source: PLoS One. 2026 Sep 21;21(9):e0358951. doi: 10.1371/journal.pone.0358951 (PMC13592612; doi:10.1371/journal.pone.0358951)
Supplement: Supplementary file 2 [file pone.0358951.s003.docx]

**S1 Text. Interview Coding Scheme and Excerpts (DOCX)**

**--Interview Excerpts and Coding Scheme**

1. Interview Participants and Data Overview

At the time of the study, none of the interview participants had prior experience with virtual simulation or systematic video analysis training. However, during the semester, all students had completed a one-day kindergarten observation practicum as part of their program’s required fieldwork, providing them with basic familiarity with classroom contexts.

After all workshops were completed, nine voluntary participants (7 females, 2 males) were recruited from the 46 preschool teacher candidates who participated in the training. Semi-structured one-on-one interviews were conducted via online voice calls, with an average duration of 20 minutes per interview. All interviews were audio-recorded with participants’ consent and transcribed verbatim, yielding approximately 25,689 words of raw text. All participants provided informed consent and were assured that their responses would be used solely for research purposes. To protect participant privacy, all names have been replaced with codes (S1–S9), and any identifiable personal information has been removed.

Data saturation was reached after the seventh interview; two additional interviews were conducted to confirm that no new themes emerged.

2. Coding Framework Overview

Through reflexive thematic analysis of the full interview corpus, a coding system was developed comprising 3 core themes, 9 cluster codes, and 26 open codes. The coding framework is presented below.

| **Core Theme** | **Cluster Code** | **Representative Open Codes** |
| --- | --- | --- |
| Theme 1: The Challenge of Finding Meaning in Chaos | Difficulties in identification and judgment | Insufficient information in VS; ambiguous observation targets in AV; difficulty determining the nature of behaviors |
|  | Difficulties in information processing | Excessive irrelevant distractors; needing multiple viewings to clarify logic; fragmented information |
|  | Difficulties in process management | Intensive workshop schedule hindering absorption; lack of practical methodological guidance |
| Theme 2: Aspirations for Structured Support | Demand for standardized support | Desire for clearer pre-observation guidance; need for behavioral checklists; expectation of clear procedures |
|  | Need for presentation optimization | VS content should be richer; AV footage quality should be more stable; supplementary background information needed |
|  | Demand for improved efficiency | Hope for more professional analysis from instructors; methodological guidance to accelerate mastery |
| Theme 3: Awareness of One's Own Growth | Enhancement of professional competence | More detailed observation; improved adaptability; enhanced practical skills |
|  | Deepening of cognitive understanding | Understanding frontline teaching scenarios; recognizing teacher roles; awareness of the theory-practice gap |
|  | Accumulation of practical experience | Early familiarization with kindergarten work contexts; gaining experience in behavioral recording and analysis |

3. Core Themes and Representative Interview Excerpts

3.1 Theme 1: The Challenge of Finding Meaning in Chaos

This theme reflects the cognitive conflict and adaptation difficulties students experienced when transitioning from virtual simulation to authentic video contexts.

3.1.1 Difficulties in Identification and Judgment

The primary challenge students faced in the VS condition stemmed from the crude presentation and lack of information, which made it difficult to accurately identify observation targets and interpret behavioral intentions:

“I feel that those virtual videos are sometimes rough. Sometimes the text does

not match the images in the video, the subtitles are inconsistent, and sometimes

you simply cannot see anything in the video, so you have to rely on the subtitles

to analyze.” (S2)

In the AV condition, the challenge originated from the ambiguity of observation targets and the polysemy of behaviors:

“In real-life situations, there is no teacher explaining the subject in advance or

providing a brief summary of what is happening. Sometimes, just by watching

the video, it is unclear who exactly is the main subject of observation.” (S2)

3.1.2 Difficulties in Information Processing

In the AV condition, information was often disorganized with numerous irrelevant distractors, requiring students to expend considerable effort to filter out effective information:

“In AV, you have to summarize on your own; it's not as simple and clear as the

virtual videos. You might need to watch them several times to figure out the logic. The videos cover multiple themes, which require careful identification, and sometimes mistakes can happen.” (S8)

3.1.3 Difficulties in Process Management

Students also faced challenges related to the intensive workshop schedule and lack of practical methodological guidance:

“The four workshops were too intensive [...] This time, we learned all the

methods first and then did the workshops. Points I didn't pay attention to at the beginning could only be patched up later.” (S9)

3.2 Theme 2: Aspirations for Structured Support

This theme reflects students' diverse needs for more refined scaffolding support after experiencing cognitive conflict.

3.2.1 Demand for Standardized Support

Students generally expressed a need for clearer pre-observation guidance and preparatory materials to clarify observation foci, procedures, and methods:

“Before the observation, we could be given clearer instructions.” (S3)

“Could the materials be sent out a few days in advance so that we have time to choose the observation methods we want to use?” (S7)

During observation, students needed standardized tools and process guidance:

“For certain observation materials and videos, we could be provided with other behavior checklists or similar tools. We could prepare checklists or charts in advance and think about whether to use them, rather than only relying on continuous real-time recording.” (S7)

3.2.2 Need for Presentation Optimization

Students articulated specific demands for improving video production quality and supplementary information:

“The virtual content could be slightly richer, with more presentation of children's behaviors and speech, to make the content more substantial.” (S1)

“The footage of AV can be shaky, making the presentation somewhat fragmented [...] Additionally, I hope supplementary background information could be provided, such as the children's age group and the context of the event, to avoid abruptness that leaves us without a starting point.” (S7)

3.2.3 Demand for Improved Efficiency

Students hoped instructors would provide more professional analysis to help them master observation methods more quickly:

“After each analysis, the teacher could, from a professional perspective, help us analyze the children's behaviors further, integrating relevant theories to explain the reasons behind those behaviors. This would help us master observation and analysis methods more quickly and improve efficiency.” (S8)

3.3 Theme 3: Awareness of One's Own Growth

This theme reflects students' positive changes in professional competence, cognitive understanding, and practical experience after participating in the sequential integration training.

3.3.1 Enhancement of Professional Competence

Students perceived notable improvements in observational skills and adaptability:

“Watching AV allows me to observe better and in more detail. At the beginning, I did not pay attention to small details, but later, influenced by my group peers, I started to notice more details, and my observational skills improved." (S8)

“Observing authentic contexts helps improve our adaptability. In authentic contexts, children's thoughts are changeable and their behaviors are flexible, so teachers need to respond flexibly. This reminds me that as a future preschool teacher, I need to improve my adaptability and learn to flexibly respond to children's various behaviors.” (S5)

3.3.2 Deepening of Cognitive Understanding

Students developed a deeper understanding of authentic kindergarten teaching scenarios, characteristics of children's behaviors, teacher roles, and the relationship between theory and practice:

“Through AV, I can gain a more intuitive understanding of frontline teaching scenarios and see children's authentic reactions more clearly.” (S8)

“When watching AI-based VS videos, I tended to focus more on the teacher’s role—how the teacher responded to children's behaviors. When watching AV, I paid more attention to the children's performance and feedback. Through this comparison, I gained a clearer understanding of the teacher's role as a guide and responder in children's activities.” (S1)

“Through observation and kindergarten practicum experiences, I realized that there is a gap between the knowledge I learned and the actual work, and I gained a deeper understanding of the importance of integrating theory and practice.”(S6)

It is worth noting that S6’s reference to practicum experiences drew on her prior fieldwork completed before the intervention, rather than experiences gained during the workshops themselves.

3.3.3 Accumulation of Practical Experience

Through participation in the observation activities, students accumulated substantial practical experience in observing children's behaviors and understanding teachers' response strategies:

“Observation in authentic contexts is crucial, as it prepares you for future work and internships.” (S2)

“Through the workshop, I understood the knowledge structure of observation, learned how to do observational records and think about support strategies, and accumulated experience in analyzing children's behaviors.” (S7)

4. Coding Process

This study employed a reflexive thematic analysis approach to code the interview data. The coding process followed these steps:

Initial coding. Two researchers independently read all interview transcripts and conducted open coding on each meaningful unit, generating 26 initial open codes.

Cluster coding. The researchers grouped conceptually similar open codes into categories, forming 9 cluster codes (as presented in the framework above).

Core theme extraction. Cluster codes were further integrated to distill 3 core themes, which captured students’ complete experiential trajectory from “cognitive conflict” to “support needs” to “perceived growth”.

Consensus building. During the coding process, the two researchers regularly discussed disagreements and reached consensus through negotiation. When consensus could not be achieved, a third senior qualitative research scholar was consulted to provide an external perspective until a unified coding framework was established.

Researcher reflexivity. We acknowledge that no formal memo-writing procedures were implemented during this study. However, we addressed potential researcher subjectivity through several complementary mechanisms. First, the two coders independently analyzed the interview data before comparing their interpretations, and regular team discussions were held to examine whether coding decisions were grounded in participant responses rather than researchers' prior assumptions. Second, when disagreements arose, a third researcher was consulted to provide an external perspective. Third, during the analysis process, the research team documented emerging interpretations and engaged in debates over alternative readings of participant responses. Although we did not produce formal written memos, these reflexive dialogues within the research team functioned as a continuous quality-check mechanism to maintain analytic transparency.

5. Data Saturation

After the seventh interview, no new open codes or core themes emerged. Two additional interviews (the eighth and ninth) confirmed the stability of the existing coding framework, with no new themes or significant deviations from the established patterns. Accordingly, qualitative data saturation was determined to have been achieved.

6. Ethical and Privacy Protection Statement

This study was approved by the University Committee on Human Research Protection at East China Normal University (Approval No. HR178-2019). All participants voluntarily enrolled in the study and provided informed consent after being fully informed of the research purpose and procedures. Participants were aware that their interview responses would be used for research publication.

Any identifiable personal information (including names, institution names, and specific class details) mentioned during the interviews has been removed or replaced with codes. All audio recordings and transcribed materials are stored on password-protected devices accessible only to the research team. This excerpt document does not contain complete interview transcripts; it only provides representative illustrative quotations related to the research themes, in order to protect participant privacy.
