# Supplementary material for: A sequential integrating virtual simulation and authentic video for preschool teacher candidates’ observational skills training: A design-based intervention study
Source: PLoS One. 2026 Sep 21;21(9):e0358951. doi: 10.1371/journal.pone.0358951 (PMC13592612; doi:10.1371/journal.pone.0358951)
Supplement: Supplementary file 3 [file pone.0358951.s005.docx]

**S2 Table. Workshop Protocol (DOCX)**

**-Workshop Protocol**

Workshop Protocol: Sequential Integration of Virtual Simulation and Authentic Video for Preschool Teacher Candidates' Observational Skills Training

--An Intervention Study Based on the Child Behavior Observation Course

1. Protocol Background and Objectives

This protocol was designed within the framework of the Child Behavior Observation course at the School of Teacher Education, Hebei Normal University. It aims to systematically enhance preschool teacher candidates' (TCs) observational skills through a sequential integration model in which virtual simulation (VS) precedes authentic video (AV). The protocol is grounded in Cognitive Load Theory (CLT) and Situated Learning Theory (SLT), and consists of four progressive workshops that transition from simplified contexts to complex, authentic classroom settings.

2. Participants

- University instructors and researchers
- 46 preschool TCs (38 females, 8 males), divided into 9 fixed groups
- One frontline kindergarten teacher (invited for an online sharing session in Workshop 2)

3. Implementation Period and Format

- Period: A 6-week teaching cycle (two sessions per week, 90 minutes per session), comprising four core workshops.
- Format: Primarily on-site, with supplementary online components (Workshop 2 included a frontline teacher's online sharing session).

4. Workshop Procedures

Table 1. Summary of the Four Sequential Workshops

| Workshop | Workshop 1 | Workshop 2 | Workshop 3 | Workshop 4 |
| --- | --- | --- | --- | --- |
| Week(s) | Weeks 1-2 | Weeks 3-4 | Weeks 5 | Weeks 6 |
| Video Type | Virtual Simulation (VS) | Authentic Video (AV) | Authentic Video (AV) | Authentic Video (AV) |
| Theme/Scenario | Peer conflict in the block construction area (middle kindergarten class) | Peer conflict during self-directed reading (middle kindergarten class) | Separation anxiety (lower kindergarten class) | Role-playing (upper kindergarten class) |
| Core Student Activities | View VS video (3-5 min) → Group discussion (20 min) → Write analytical text (40 min) | View AV video (3-5 min) → Group discussion → Write analytical text | View AV video → Group discussion → Write analytical text (analysis dimension only) | View AV video → Group discussion → Write complete analytical text |
| Data Collection | Collect group analytical texts; administer Questionnaire T1 | Collect group texts; administer Questionnaire T2 | Collect group texts (analysis dimension only) | Collect group texts; administer Questionnaire T3; conduct semi-structured interviews after workshops (voluntary, n=9) |
| Instructor/Expert Support | Instructor provides three-dimensional feedback (theoretical perspectives, historical factors, environmental context) | Instructor three-dimensional feedback; Additional support: Frontline kindergarten teacher online sharing session (approx. 20 min) | Instructor three-dimensional feedback | Instructor three-dimensional feedback; research team summary |

Notes to Table 1:

- All videos were 3–5 minutes in duration. VS materials were independently developed by the research team using the Doubao which supports end-to-end generation of simulation-grade 3D models from textual prompts. AV materials were provided by a collaborating kindergarten (recorded in daily classroom settings), with children's faces anonymized using mosaic processing.
- The teacher sharing session in Workshop 2 constituted additional support and may have influenced student performance in the AV condition; this is acknowledged as a potential confounding factor in the Discussion (Section 5.1) and Limitations (Section 6.2) of the main text.
- The three questionnaire time points were: T1 (after Workshop 1), T2 (after Workshop 2), and T3 (after Workshop 4), used to track changes in students' self-perceived observation knowledge.

5. Scoring Rubric for Group Observation Assignments

Following each workshop, each group submitted an analytical text. The texts were scored based on three core dimensions (total score = 100 points):

| Dimension | Points | Assessment Criteria |
| --- | --- | --- |
| Observation Dimension | 40 | Whether students could objectively and accurately describe children's behaviors, attending to contextual factors such as physical environment, peer interactions, and teacher involvement |
| Analysis Dimension | 30 | Whether students could interpret children's behaviors by applying relevant theories (e.g., child development, psychology) |
| Support Dimension | 30 | Whether students could propose actionable educational support suggestions by integrating children's prior experiences and developmental needs |

Detailed level-specific descriptors for each performance level are provided in Supplemental Table S1.

6. Data Collection and Ethical Statement

- Quantitative data: Group analytical text scores (32 valid samples); three waves of questionnaires (34 students with complete participation across all three time points).
- Qualitative data: Semi-structured interviews with 9 students (voluntary sign-up; audio recordings transcribed verbatim, yielding approximately 25,689 words of text).
- Ethical approval: This study was approved by the University Committee on Human Research Protection at East China Normal University (Approval No. HR178-2019). All adult participants provided informed consent. Children's faces in all authentic videos were anonymized to protect privacy.
